# Bistratamides M and N, Oxazole-Thiazole Containing Cyclic Hexapeptides Isolated from *Lissoclinum bistratum* Interaction of Zinc (II) with Bistratamide K

**DOI:** 10.3390/md15070209

**Published:** 2017-07-01

**Authors:** Carlos Urda, Rogelio Fernández, Jaime Rodríguez, Marta Pérez, Carlos Jiménez, Carmen Cuevas

**Affiliations:** 1 Medicinal Chemistry Department, PharmaMar S. A., Polígono Industrial La Mina Norte, Avenida de los Reyes 1, 28770 Madrid, Spain; curda@pharmamar.com (C.U.); rfernandez@pharmamar.com (R.F.); ccuevas@pharmamar.com (C.C.); 2 Department of Chemistry, Faculty of Sciences and Center for Advanced Scientific Research (CICA), University of A Coruña, 15071 A Coruña, Spain; jaime.rodriguez@udc.es

**Keywords:** cytotoxic, sponge, *Lissoclinum bistratum*, cyclic hexapeptides, bistratamides, Zn complex

## Abstract

Two novel oxazole-thiazole containing cyclic hexapeptides, bistratamides M (**1**) and N (**2**) have been isolated from the marine ascidian *Lissoclinum bistratum (L. bistratum)* collected in Raja Ampat (Papua Bar, Indonesia). The planar structure of **1** and **2** was assigned on the basis of extensive 1D and 2D NMR spectroscopy and mass spectrometry. The absolute configuration of the amino acid residues in **1** and **2** was determined by the application of the Marfey’s and advanced Marfey’s methods after ozonolysis followed by acid-catalyzed hydrolysis. The interaction between zinc (II) and the naturally known bistratamide K (**3**), a cyclic hexapeptide isolated from a different specimen of *Lissoclinum bistratum*, was monitored by ^1^H and ^13^C NMR. The results obtained are consistent with the proposal that these peptides are biosynthesized for binding to metal ions. Compounds **1** and **2** display moderate cytotoxicity against four human tumor cell lines with GI_50_ values in the micromolar range.

## 1. Introduction

Ascidians of the genus *Lissoclinum* are a rich source of cyclic peptides, many of which incorporate modified amino acid residues containing thiazole, oxazole, thiazoline, or oxazoline rings. Examples reported in the literature include the hexapeptides bistratamides A–J [1,2,3], cycloxazoline [4] (also known as westiellamide) [5], the heptapeptides nairaiamides A and B [6], and lissoclinamides 4 and 5 [7], and the octapeptides patellamides A–C [8] and tawicyclamides A and B [9]. 

The symbiotic microbial origin of the cyclic peptides isolated from specimens of the genus *Lissoclinum* has been proposed based on the fact that similar structures have been discovered from cyanobacteria [10]. Ascidians harbor an obligate symbiont, *Prochloron* sp., a cyanobacterium that photosynthesizes nutrients for the sea squirt, which is thought to be involved in the biosynthesis of the cyclic peptides as secondary metabolites [11]. This hypothesis has been confirmed for the case of the patellamides [12] and an efficient method for the in vivo production of this type of cyclic peptide has also been described [13].

Some of these azole-based cyclic peptides and their synthetic derivatives have shown antibacterial, antiviral, or cytotoxic activities, along with metal binding properties. In fact, the concentration of metal ions such as Cu^2+^ and Zn^2+^ in ascidian cells has been found to reach values over 104 times those detected in the surrounding sea water [14]. The necessary structural and stereochemical features to facilitate metal complexation along with the biological relevance of the metal ions and their possible role in the assembly of cyclic peptides in the marine environment have been proposed [15]. Moreover, the former biological activities could be attributable to the conformational constraints imposed by the heterocycles and their ability to bind metals or intercalate into DNA. Particularly, the antitumor activities and the potential to act as metal ion chelators have made these azole-based cyclic peptides attractive targets for total synthesis and biological evaluation [16].

As part of our ongoing efforts to find novel antitumor agents from marine organisms and specifically from ascidians [17,18], a detailed biological investigation of a specimen of *L. bistratum* collected by hand off the coast of Raja Ampat Islands, Indonesia, showed that its organic extract displayed cytotoxic activity against the human tumor cell lines A-549 (lung), HT-29 (colon), MDA-MB-231 (breast) and PSN1 (pancreas). Bioassay-guided fractionation of the active organic extract resulted in the isolation of two new cyclic hexapeptides, bistratamides M (**1**) and N (**2**), which show significant cytotoxicity towards different human cancer cells.

## 2. Results and Discussion

### 2.1. Isolation and Structure Elucidation

**Figure 1:**
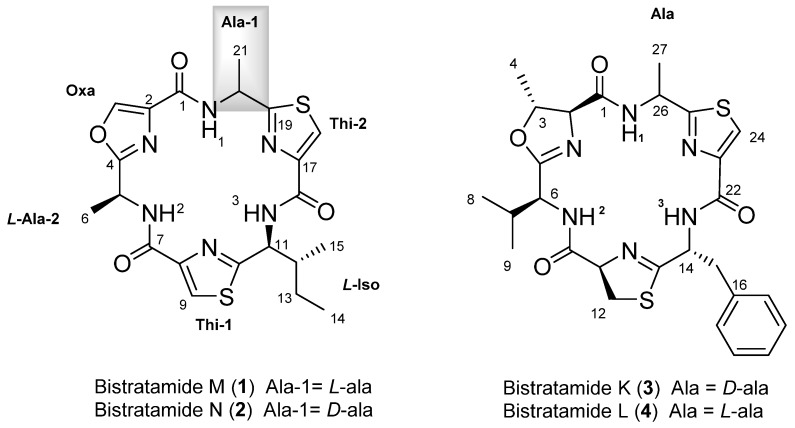
Chemical structures of compounds **1**–**4**.

A specimen of the marine ascidian *L. bistratum* was extracted several times using CH_2_Cl_2_/MeOH (1:1). The extract was subsequently fractionated by vacuum flash chromatography (VFC) on a Lichoprep RP-18 column using a gradient mixture of H_2_O, MeOH and CH_2_Cl_2_ with decreasing polarity. Bioassay-guided isolation using the previously described human tumour cell lines yielded a very active fraction (eluted with 100% MeOH) that was subjected to reversed-phase HPLC to yield **1** and **2** (Figure 1).

Bistratamide M (**1**) was obtained as a colourless amorphous solid with a molecular formula C_21_H_24_N_6_O_4_S_2_ (13 degrees of unsaturation) determined by the [M + H]^+^ ion peak at *m*/*z* 489.1405, detected in its (+)-HRESI-TOFMS. The hexapeptide structure of **1** was suggested by the six nitrogen atoms present in its molecular formula along with the six sp^2^ carbon signals between *δ*_C_ 159.4 and 171.6 observed in its ^13^C NMR spectrum (Table 1). The presence of only three amide NH signals at *δ*_H_ 8.42, 8.64, and 8.69, along with three singlet aromatic protons at *δ*_H_ 8.12, 8.22, and 8.27, observed in the ^1^H NMR spectrum of **1**, suggested the existence of three cyclically-modified amino acids.

2D NMR experiments of **1**, including COSY, TOCSY, and edited HSQC, allowed us to identify their amino acid residues. Thus, the diagnostic methyl groups at *δ*_H_ 1.72 (d, 7.1)/*δ*_C_ 19.9 and *δ*_H_ 1.74 (d, 6.9)/*δ*_C_ 23.9 were indicative of the presence of two alanines, while the spin system *δ*_H_ 8.42 (d, *J* = 8.0 Hz, NH), 5.44 (m, 1H)/2.18 (m, 1H)/1.63, 1.24 (m, 2H)/1.01 (t, *J* = 7.4 Hz, 3H), and 0.87 (d, *J* = 6.8 Hz, 3H) showed the existence of an isoleucine residue. Additionally, the proton and carbon NMR aromatic signals at *δ*_H_ 8.12 (s, 1H)/*δ*_C_ 123.0 and at *δ*_H_ 8.22 (s, 1H)/*δ*_C_ 125.0 suggested the occurrence of two thiazole rings while those at *δ*_H_ 8.27 (s, 1H)/*δ*_C_ 141.9 indicated the existence of an oxazole moiety. These rings are the result of the condensation of two cysteines and one serine residue, respectively (Table 1). The former assignments accounted for 12 of the 13 unsaturation degrees required by the molecular formula, so the cyclic nature of this hexapeptide was deduced from the remaining unsaturation. 

**Figure 2:**
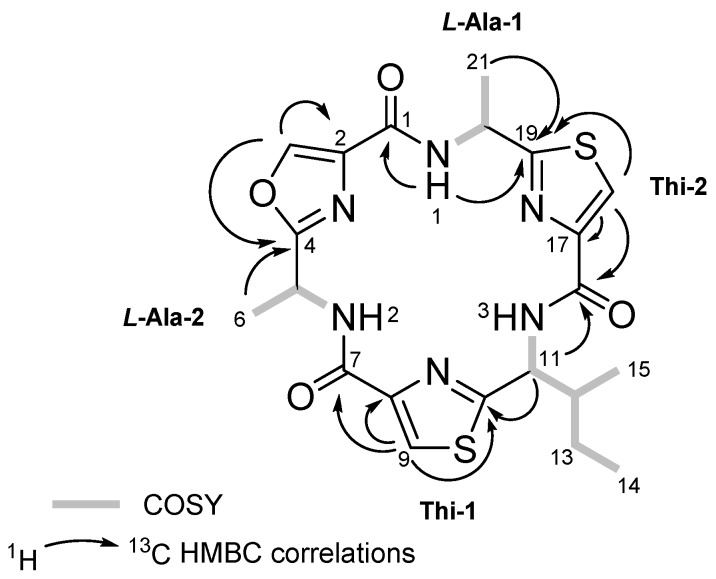
COSY correlations (displayed by bold bonds) and key HMBC (arrows) in **1** and **2**.

The long range proton and carbon correlations observed in the HMBC experiment of **1** from each of the amide NH protons allowed to us to establish not only the amino acid sequence of this cyclic peptide but also to confirm the presence of the thiazole and oxazole rings. Thus, the HMBC correlations from the alanine amide NH proton at *δ*_H_ 8.69 (NH-1) to C-19 at *δ*_C_ 171.6, corresponding to the thiazole-2 ring, and to carbonyl C-1 at *δ*_C_ 159.7, corresponding to the oxazole ring, allowed us to locate the first alanine residue (Ala-1) between the thiazole (Thi-2) and oxazole rings. The location of the isoleucine residue between the two thiazole rings was deduced from the long range correlations from its α-proton at *δ*_H_ 5.44 (H-11) to C-10 at *δ*_C_ 167.1 and to C-16 at *δ*_H_ 159.8, assigned to the thiazole-1 and thiazole-2 aromatic rings, respectively. Finally, the HMBC correlation between the oxazole aromatic proton at *δ*_H_ 8.27 (H-3) to C-2 at *δ*_C_ 135.5 and to C-4 at *δ*_C_ 164.3 of the oxazole ring and this in turn to H-6 at *δ*_H_ 1.72 of the second alanine established the link between Ala-2 and the oxazole ring (Figure 2). Following the established nomenclature used for the family of cyclic hexapeptides isolated from *L. bistratum*, compound **1** was named as bistratamide M.

To complete the structure of **1**, the absolute configuration of the amino acids was established via acid hydrolysis of the product obtained by ozonolysis of **1** followed by derivatization with Marfey’s reagent and posterior analysis by HPLC. Thus, the HPLC analysis of l-FDAA (Marfey’s method) for the alanines [19] and l-FDAA and d-FDAA derivatives for the isoleucine (advanced Marfey’s method) [20,21] showed that **1** contains two l-alanine and one l-isoleucine residues.

Bistratamide N (**2**) was obtained as colourless amorphous solid. Its molecular formula, C_21_H_24_N_6_O_4_S_2_, determined by (+)-HRESI-TOFMS, proved to be the same as **1**. Moreover, the proton and carbon NMR chemical shifts of both compounds were very similar, suggesting that **2** contained the same residues but different stereochemistry for at least one of the amino acid residues. Analysis of the HMBC data of **2**, showing similar HMBC correlations to those of **1**, displayed the same amino acid sequence as in its isomer **1**. Indeed, the long range correlations between the first alanine methyl protons at *δ*_H_ 1.75 (H-21) to C-19 at *δ*_C_ 171.0 and this in turn to the aromatic proton at *δ*_C_ 8.17 (H-18) of the thiazole (Thi-2) linked the first alaline residue to the thiazole ring. The HMBC correlations from the isoleucine α-proton at *δ*_H_ 5.54 (H-11) to C-16 (*δ*_C_ 159.8) and C-10 (*δ*_C_ 167.9) confirmed the location of the isoleucine between the two thiazole rings. Additionally, HMBC correlations from the oxazole aromatic proton at *δ*_H_ 8.23 (H-3) to C-2 (*δ*_C_ 135.6) and C-4 (*δ*_C_ 164.6) and this, in turn, to H-6 (*δ*_H_ 1.72), of the second alanine, completed the sequence assignment. The absolute configuration of **2** was obtained in a similar way as for **1**. Compound **2** was submitted to ozonolysis, followed by acid hydrolysis and derivatization with Marfey’s reagent l-FDAA (Marfey’s method) for the alanines, and l-FDAA and d-FDAA for the isoleucine (advanced Marfeýs method). HPLC-MS analysis of the resulting derivatives showed the presence of l- and d-alanine and l-isoleucine. Thus, an additional problem in this case was to establish the exact position of each alanine residue in compound **2**. Taking into account that acid hydrolysis of this type of cyclic peptides, using 6N HCl at 110 °C, means that the oxazole rings will be cleaved whilst the thiazole rings remain intact, compound **2** was subjected to these hydrolytic conditions [22]. HPLC separation of the resulting acid hydrolysates followed by analysis using Marfey’s method showed the presence of l-alanine instead of d-alanine. Consequently, we deduced that the alanine residue linked to the oxazole ring has the L configuration, while the alanine linked to the thiazole ring in **2** must have the D configuration.

The proton and chemical shifts of bistratamides M and N (**1** and **2**) are similar to those of the reported for dolastatin E which was isolated from the sea hare *Dolabella auricularia*. They differ in one of the heterocycle rings: the second thiazole ring (Thi-2) in **1** and **2** is replaced by a thiazoline moiety in dolastatin E. However, the absolute stereochemistry of the aminoacid residues in dolastatin E was not determined [23].

### 2.2. Interaction Studies of Zinc (II) with Bistratamide K

The extremely low level of biologically-available essential metals in the marine environment suggests that marine organisms have developed unique mechanisms for their acquisition, retention, and utilization. Little is known about these mechanisms, but it has been suggested that secondary metabolites may play a vital role. Many marine secondary metabolites contain functional groups that can complex metals, but there is a lack of evidence as to whether this occurs in vivo [24]. Furthermore, an improved understanding of the role of metal ions is required not only for studying the mechanism of enzymatic catalysis by metalloenzymes, but also because transition metal ions play an important role in pathophysiological processes and the perturbation of zinc-finger binding. As such, small peptides such as the bistratamides are very useful model compounds for the study of complex formation.

**Figure 3:**
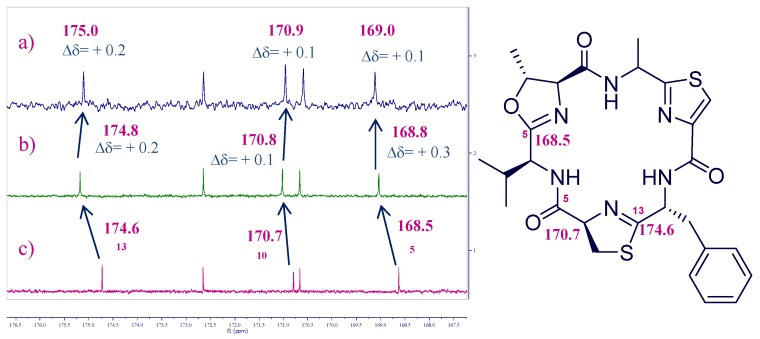
Partial ^13^C NMR spectra of compound **3** after addition of a ZnCl_2_ solution: (**a**) 4 equiv.; (**b**) 2 equiv.; and (**c**) 0 equiv. in CD_3_CN.

**Figure 4:**
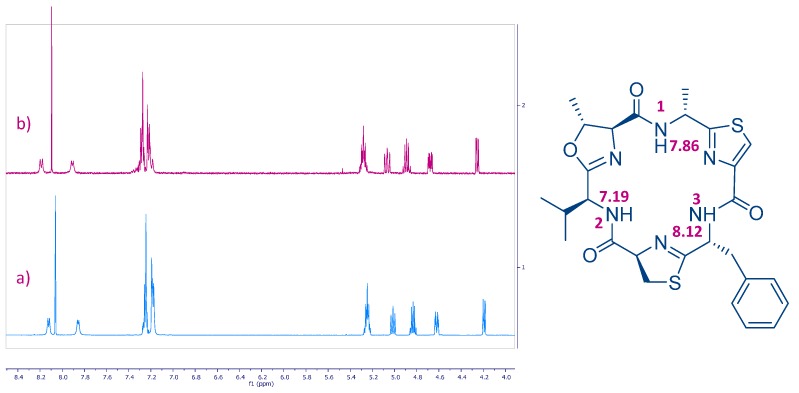
Partial ^1^H NMR spectra of (**a**) compound **3** and (**b**) after addition of a 4 equiv. ZnCl_2_ solution in CD_3_CN.

Azole-based cyclic peptides found in ascidians of the genus *Lissoclinum* have a high propensity to chelate metal ions. Although there are many studies on metal binding of azole-based cyclic octapeptides, those related to cyclic hexapeptides [25,26] are less common [27]. In order to study the chelating properties of this type of oxazole-thiazole cyclic hexapeptides, we focused our attention on bistratamide K (**3**), also isolated by our research group at PharmaMar in a reasonable amount along with its l-alanine isomer, bistratamide L (**4**) (Figure 1), from another specimen of *Lissoclinum* of the same expedition [28]. Initial trials of the interactions of copper (II) and lithium with **3** were unsuccessful. However, interesting results were obtained when we tested the interaction of Zn (II) with **3**. The Zn (II)-binding behaviour of **3** was studied in CD_3_CN by adding a ZnCl_2_ solution to a solution of the peptide in a NMR tube and analysis of the resulting ^13^C and ^1^H NMR spectra. Spectral changes were observed in the carbon chemical shifts of the ^13^C NMR spectra of **3** after addition of 2 and 4 equivalents of ZnCl_2_. The downfield carbon chemical shifts at positions C-5 (*δ*_C_ 168.5), C-10 (*δ*_C_ 170.7) and C-13 (*δ*_C_ 174.6) suggested that these positions are involved in the zinc binding of **3** (Figure 3). However, no changes were observed in the corresponding ^1^H NMR spectrum (Figure 4), which seems to indicate that none of the amide nitrogen atoms is bound to the metal (the corresponding NH proton chemical shift signals neither moved nor disappeared).

This result contrasts to that obtained by Comba et al. in the coordination studies of Cu^2+^ ions with westiellamide, a similar cyclic hexapeptide, and three synthetic analogues [29]. Their corresponding mononuclear complexes showed N_het_-N_amide_-N_het_ binding sites and each Cu^2+^ ion was coordinated by three nitrogen atoms of the macrocycle, two of the nitrogen donors originating from azole rings and the third one from an NH amide group. The coordination sphere was completed by solvent molecules. In our case, deprotonation of the amide nitrogen donor is not observed and so, the Zn^2+^ ion is not coordinated by an amide nitrogen atom. The changes seen in positions C-5, C-10, and C-13 are in agreement with the studies carried out with Gahan et al., whereby the K^+^ complex of the cyclic octapeptide ascidiacyclamide showed that the potassium ion was bound to the two nitrogen atoms of the thiazole rings and to the oxygen center of the adjacent amide groups [30].

**Figure 5:**
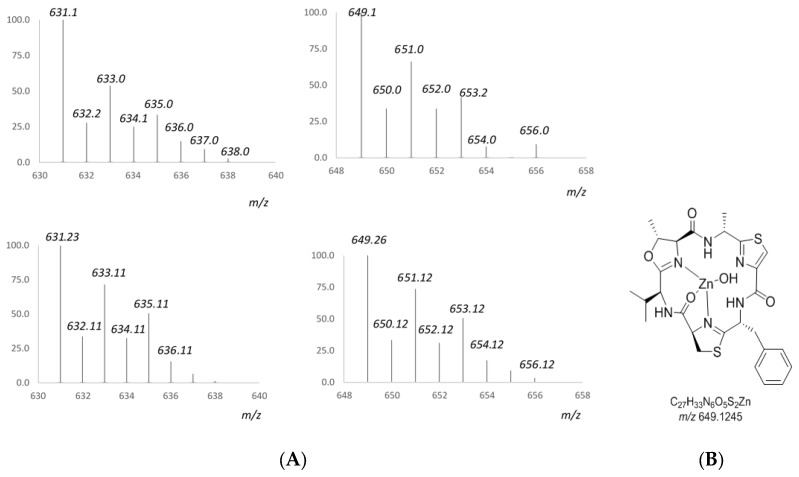
(**A**) Experimental (**top**) and calculated (**bottom**) isotopic clusters of compound **3** after addition of a 4 equiv. ZnCl_2_ solution detected in its (+)-ESI-TOFMS. (**B**) Structure proposal for a mononuclear Zn^2+^ complex of **3**.

A further study of the zinc complex of **3** by mass spectrometry was carried out in order to ascertain its structure. Analysis of isotope patterns were employed to assign the components of the complex. The main cluster ions detected in the (+)-ESI-TOF mass spectrum of **3** (see Supplementary Materials) after addition of 4 equiv. of Zn^2+^ along with the corresponding calculated isotopic patterns are displayed in Figure 5. Thus, the ion cluster observed at *m/z* 649 was assigned to a mononuclear Zn^2+^ complex of **3** which is further coordinated to an OH group. The loss of 18 units, giving the ion cluster detected at *m/z* 631 as the base peak, could be due to the loss of a molecule of water.

Cell proliferation assays against the human tumor cell lines MDA-MB-231 (breast), HT-29 (colon), NSLC A-549 (lung), and PSN1 (pancreas), and showed that both **1** and **2** exhibit moderate cytotoxic activity with GI_50_ values in the micromolar range (Table 2). As a positive standard the antitumor compound doxorubicin was also tested in parallel following an identical procedure and the results are included in Table 2. These cytotoxic activity values are similar to those reported for cyclic hexapeptides bistratamides E–J. Interestingly, it has been found that cyclic peptides containing two thiazole rings instead of one thiazole and one oxazole ring display higher activity [3,31]. In order to get some insights into the mechanism of action of the cytotoxic activity of these compounds, a further study was performed. Compounds **1** and **2** were tested in the enzymatic Topoisomerase 1 (Top1) assay using human recombinant enzyme as described in the experimental section, and failed to show any hint of inhibition even at the highest concentration tested (100 μM).

## 3. Materials and Methods

### 3.1. General Experimental Procedures

Optical rotations were determined using a Jasco P-1020 polarimeter (Oklahoma City, OK, USA). UV spectra were performed using an Agilent 8453 UV-VIS spectrometer (Santa Clara, CA, USA). IR spectra were obtained with a Perkin-Elmer Spectrum 100 FT-IR spectrometer (Waltham, MA, USA) with ATR sampling. NMR spectra were recorded on a Varian “Unity 500” spectrometer (Palo Alto, CA, USA) at 500/125 MHz (^1^H/^13^C). Chemical shifts were reported in ppm using residual CDCl_3_ (*δ* 7.26 ppm for ^1^H and 77.0 ppm for ^13^C) and CD_3_CN (*δ* 1.96 ppm for ^1^H, and 118.3 and 1.8 ppm for ^13^C) as an internal reference. (+)-ESIMS were recorded using an Agilent 1100 Series LC/MSD spectrometer. High-resolution mass spectroscopy (HRMS) was performed with an Agilent 6230 TOF LC/MS system using the ESI-MS technique.

### 3.2. Animal Material

The ascidian *Lissoclinum bistratum* was collected by hand and traditional scuba diving in Raja Ampat Islands (Papua Bar, Indonesia) (00° 33.353′ S/130° 41.156′ E) at depths ranging between 1 and 8 m in April 2007 and frozen immediately after collection. A voucher specimen (ORMA48136) is deposited at PharmaMar.

### 3.3. Extraction and Isolation

A specimen of *Lissoclinum bistratum* (75 g) was triturated and exhaustively extracted with CH_3_OH:CH_2_Cl_2_ (50:50, 3 × 500 mL). The combined extracts were concentrated to yield a crude mass of 2.6 g that was subjected to VLC on Lichroprep RP-18 (Merck KGaA, Kenilworth, NJ, USA) with a stepped gradient from H_2_O to CH_3_OH and then CH_2_Cl_2_. The fraction eluting with CH_3_OH (351 mg) was subjected to semi-preparative HPLC (Waters XBridge C_18_, 5 μm, 10 × 150 mm, gradient from 33 to 73% CH_3_CN in H_2_O with 0.1% TFA in 20 min, flow: 5 mL/min, UV detection, Milford, MA, USA) to yield **1** (6 mg, retention time: 15.1 min) and **2** (3.4 mg, retention time: 9.5 min). 

Bistratamide M (**1**): Compound **1** was isolated as an amorphous, colorless solid; [α]${}_{D}^{25}$ −40.2 (*c* 1.4, MeOH); UV (MeOH) *λ*_max_ 202 nm; IR (ATR) *ν*_max_ 3396, 2969, 2932, 1678, 1604, 1496, 1208, 1142, 845, 802, 726 cm^−1^; ^1^H NMR (500 MHz) and ^13^C NMR (125 MHz) see Table 1; (+)-HRESI-TOFMS *m/z* 489.1405 [M + H]^+^ (calcd. for C_21_H_25_N_6_O_4_S_2_, *m/z* 489.1373).

Bistratamide N (**2**): Compound **2** was isolated as an amorphous, colorless solid; [α]${}_{D}^{25}$ −9.6 (*c* 1.1, MeOH); UV (MeOH) *λ*_max_ 202 nm; IR (ATR) *ν*_max_ 3395, 2969, 2932, 1678, 1604, 1541, 1496, 1208, 1143, 845, 803, 726 cm^−1^; ^1^H NMR (500 MHz) and ^13^C NMR (125 MHz) see Table 1; (+)-HRESI-TOFMS *m/z* 489.1383 [M + H]^+^ (calcd. for C_21_H_25_N_6_O_4_S_2_, *m/z* 489.1373).

### 3.4. Absolute Configuration

Bistratamides M (**1**) (0.2 mg, 0.4 mmoL) and N (**2**) (0.2 mg, 0.4 mmoL) were dissolved in dry CH_2_Cl_2_ (3 mL), and a stream of ozone in oxygen was bubbled through each solution for 5 min. After solvent removal under vacuum, the resulting crude products were hydrolyzed in 0.5 mL of 6 N HCl at 110 °C for 18 h. Excess aqueous HCl was removed under a N_2_ stream and 100 μL of H_2_O, 0.4 mg of 1-fluoro-2,4-dinitrophenyl-5-l-alaninamide (l-FDAA, Marfey’s reagent) in 100 μL of acetone and 1 M NaHCO_3_ (40 μL) was added to the dry hydrolysates. The resulting mixtures were heated at 40 °C for 1 h. Then, the reaction mixtures were cooled to 23 °C, quenched by addition of 2 N HCl (100 μL), dried, and dissolved in H_2_O (660 μL). Each aliquot was then subjected to reversed-phase LC/MS (column: Waters Symmetry, 150 × 4.6 mm, 5 μm; mobile phase, CH_3_CN + 0.04% TFA/H_2_O + 0.04% TFA; flow rate, 0.8 mL/min) using a linear gradient (20–50% CH_3_CN over 30 min). The retention times and ESIMS product ions (*t*_R_ in min, *m/z* [M + H]^+^) of the l-FDAA mono-derivatized amino acids in the hydrolysates of **1** and **2** were established as l-Ala (14.1, 342.2) in **1** and l-Ala (14.3, 342.2) and d-Ala (16.8, 342.2) in **2**.

Advanced Marfey’s analysis of isoleucine standard: To 0.3 mg (2.2 mmoL) of a 1:1 mixture of l-Ile and d-*allo*-Ile standards (Sigma I2877) was added a 1:1 racemic mixture of 0.2 mg of 1-fluoro-2,4-dinitrophenyl-5-l-alaninamide (l-FDAA) in 50 μL of acetone, 0.2 mg of 1-fluoro-2,4-dinitrophenyl-5-d-alaninamide (d-FDAA) in 50 μL of acetone, and 1 M NaHCO_3_ (40 μL), and the mixture was heated at 40 °C for 1 h. After that time, the mixtures were cooled to 23 °C, quenched by addition of 2 N HCl (100 μL), dried, and dissolved in H_2_O (660 μL) to obtain the four stereoisomers of isoleucine. Analysis of the retention times of the derivatized amino acids (*t*_R_ in min), using LC (Phenomenex Lux Cellulose-4 column, 250 × 4.6 mm, 5 μm, 100 Å; mobile phase, CH_3_CN containing 0.1% TFA; flow rate, 1 mL/min using an isocratic gradient (35% CH_3_CN over 50 min)), were l-FDAA-l-Ile (33.9), l-FDAA-d-*allo*-Ile (36.0), d-FDAA-l-Ile (31.4) (equivalent to l-FDAA-d-Ile), and d-FDAA-d-*allo*-Ile, (27.9) (equivalent to l-FDAA-l*-allo*-Ile). Comparison of *t*_R_ of the Ile unit in the hydrolysates of **1** and **2** (33.9) with the l/d-FDAA-derivatized amino acid standards, showed the configuration of Ile to be L in both cases.

### 3.5. Titration of Bistratamide K (***3***)

Three milligrams of Bistratamide K was dissolved in CD_3_CN (0.75 mL) and titrated by adding 0.2 by 0.2 equiv. of ZnCl_2_ in CD_3_CN until reaching a total of 4 equiv. NMR experiments were carried out after each 0.2 equiv. were added.

### 3.6. Biological Assays

The cytotoxic activity of compounds **1** and **2** was tested against NSLC A-549 human lung carcinoma cells, MDA-MB-231 human breast adenocarcinoma cells, HT-29 human colorectal carcinoma cells, and PSN1 (human pancreatic carcinoma cells). The concentration giving 50% inhibition of cell growth (GI_50_) was calculated according to the procedure described in the literature [32]. Cell survival was estimated using the National Cancer Institute (NCI) algorithm [33]. Three dose response parameters were calculated for **1** and **2**.

The activity of human topoisomerase I was determined based on the increase in fluorescence of a commercial dye (“H19”, Profoldin, Westborough, MA, USA) when interacting with DNA and the higher number of interaction points present in supercoiled DNA with respect to relaxed DNA. Therefore, the activity of the enzyme causes a decrease of fluorescence intensity when the probe is added. The enzyme reaction is performed in a final volume of 20 μL in 384 black wells. Compounds **1** and **2**, at twice their final concentrations, were mixed with 50 pM recombinant human topoisomerase I (Profoldin, Westborough, MA, USA) in 10 μL of 10 mM Tris-HCl pH 8.0 with 150 mM sodium chloride, 3 mM magnesium chloride and 5% (*v*/*v*) glycerol and incubated for 1 h at room temperature. Then, reaction was started by the addition of 10 μL of supercoiled DNA (Profoldin, Westborough, MA, USA) at 25 μg/mL in the same buffer and the mixture was incubated at room temperature for 1 h. Finally, the reaction was stopped by the addition of 30 μL of H19 dye in its commercial buffer to reach the dye concentration recommended by the vendor. After 1 h at room temperature the degree of conversion from supercoiled to relaxed DNA was determined by monitoring fluorescence intensity (excitation at 485 nm, emission at 535 nm) in a microtiter plate reader. Final compound concentration covered a range from 100 to 0.2 μM following 10 serials of 1:2 dilutions. IC_50_ values were calculated by fitting the results to a typical four parameters logistic curve by nonlinear regression analysis.

## 4. Conclusions

In summary, we have isolated two novel cyclic hexapeptides from the ascidian *Lissoclinum bistratum*, and bistratamides M (**1**) and N (**2**). NMR and MS analysis and Marfey’s method were used for the determination of their planar structures and absolute configurations and they are characterized by the presence of oxazole and thiazole rings. These cyclic peptides showed moderate cytotoxic activity against four human tumor cell lines. In addition, a study of the interaction of Zn^2+^ with another cyclic hexapeptide, bistratamide K (**3**), isolated from the same organism indicated the formation of a mononuclear Zn^2+^complex of **3** as shown by NMR and confirmed by ESI-TOFMS experiments. These results help provide further understanding of the relationships between metals and the azole-based cyclic peptides found in ascidians [29].

## Figures and Tables

**Table 1:** NMR data of **1** and **2** in CDCl_3_ (500 MHz for ^1^H and 125 MHz for ^13^C).

No.	Bistratamide M (1)		Bistratamide N (2)	
	*δ*_C_, Type	*δ*_H_ Mult, (*J* in Hz)	*δ*_C_, Type	*δ*_H_ Mult, (*J* in Hz)
1	159.7, C	-	159.0, C	-
2	135.5, C	-	135.6, C	-
3	141.9, CH	8.27, s	141.5, CH	8.23, s
4	164.3, C	-	164.6, C	-
5	44.2, CH	5.38, m	44.1, CH	5.37, m
6	19.9, CH_3_	1.72, d (7.1)	20.8, CH_3_	1.72, d (6.8)
7	159.4, C	-	159.5, C	-
8	149.2, C	-	149.1, C	-
9	123.0, CH	8.12, s	123.3, CH	8.12, s
10	167.1, C	-	167.9, C	-
11	55.3, CH	5.44, m	54.9, CH	5.54, m
12	40.1, CH	2.18, m	41.5, CH	2.09, m
13	26.3, CH_2_	1.63, m; 1.24, m	25.6, CH_2_	1.67, m; 1.32, m
14	11.5, CH_3_	1.01, t (7.4)	11.6, CH_3_	1.02, t (7.4)
15	14.5, CH_3_	0.87, d (6.8)	15.1, CH_3_	0.97, d (6.8)
16	159.8, C	-	159.8, C	-
17	148.2, C	-	148.6, C	-
18	125.0, CH	8.22, s	124.3, CH	8.17, s
19	171.6, C	-	171.0, C	-
20	48.2, CH	5.40, m	47.7, CH	5.46, m
21	23.9, CH_3_	1.74, d (6.9)	24.8, CH_3_	1.75, d (6.7)
NH-1	-	8.69, d (5.7)	-	8.71, d (6.5)
NH-2	-	8.64, d (7.2)	-	8.65, d (7.3)
NH-3	-	8.42, d (8.0)	-	8.46, d (9.0)

**Table 2:** Cytotoxic activity data (μM) of **1** and **2**.

Compound		Cell Line			
		Breast	Colon	Lung	Pancreas
		MDA-MB-231	HT-29	NSLC A-549	PSN1
**Bistratamide M (1)**	GI_50_	18	16.0	9.1	9.8
	TGI	>20.0	>20.0	>20.0	>20.0
	LC_50_	>20.0	>20.0	>20.0	>20.0
**Bistratamide N (2)**	GI_50_	>20.0	13.0	11.0	15.0
	TGI	>20.0	>20	>20.0	>20.0
	LC_50_	>20.0	>20	>20.0	>20.0
**Doxorubicin**	GI_50_	0.2	0.3	0.2	0.2
	TGI	0.5	0.9	0.9	0.5
	LC_50_	2.4	>17.2	>17.2	3.1

**G**I_50_, compound concentration that produces 50% inhibition on cell growth as compared to control cells; TGI, compound concentration that produces total growth inhibition as compared to control cells, and LC_50_, compound concentration that produces 50% cell death as compared to control cells.

## Supplementary Materials

The following are available online at www.mdpi.com/1660-3397/15/7/209/s1, Figures S1–S26: NMR spectra of bistratamides M, N, and K; Figure S27: Key HMBC of bistratamide K; Figures S28–S31: Analysis by Marfey’s method; Figures S32 and S33: NMR spectra data of bistratamide K after addition of ZnCl_2_ solutions; Figure S34: (+)-ESI-TOFMS of bistratamide K after addition of 4 equiv. ZnCl_2_ solution; Table S1: NMR data of bistratamides M, N, and K.
