# Supplementary material for: Bistratamides M and N, Oxazole-Thiazole Containing Cyclic Hexapeptides Isolated from Lissoclinum bistratum Interaction of Zinc (II) with Bistratamide K
Source: Mar Drugs. 2017 Jul 1;15(7):209. doi: 10.3390/md15070209 (PMC5532651; doi:10.3390/md15070209)
Supplement: Supplementary file 1 [file marinedrugs-15-00209-s001.pdf]

Supplementary Materials

Bistratamides M and N, oxazole-thiazole containing cyclic hexapeptides isolated from *Lissoclinum bistratum*. Interaction of zinc (II) with the cyclic hexapeptide bistratamide K.

Carlos Urda,[†] *Rogelio Fernández,*[†] *Jaime Rodríguez,*[‡] *Marta Pérez,*[*†] *Carlos Jiménez*[‡*] *and Carmen Cuevas,*[†]

[†] Medicinal Chemistry Department, PharmaMar S. A., Pol. Ind. La Mina Norte, Avenida de los Reyes 1, 28770, Colmenar Viejo (Madrid), Spain.

[‡] Departamento de Química, Facultade de Ciencias e Centro de Investigacións Científicas Avanzadas (CICA) Universidade da Coruña. 15071 A Coruña, Spain.

INDEX

Table S1. NMR data of **1**, **2** and **3**………………………………………………… S3
Figure S1-S13. NMR spectra data of bistratamide M (**1**) in $CDCl_3$………….. S4-S10
Figure S14-S21. NMR spectra data of bistratamide N (**2**) in $CDCl_3$……………S11-S14
Figure S22-S26. NMR spectra data of bistratamide K (**3**) in $CDCl_3$……………S15-S17
Figure S27. Structure and key HMBC of bistratamide K (**3**)……………………S17
Figure S28. LC/MS analysis of bistratamide M (**1**) by Marfey´s method using ozonolysis, hydrolysis and derivatization with L-FDAA………………………. S18
Figure S29. LC/MS analysis of bistratamide N (**2**) by Marfey´s method using ozonolysis, hydrolysis and derivatization with L-FDAA………………………..S18
Figure S30. Analysis of bistratamide M (**1**) by Advance Marfey´s method…… S19
Figure S31. LC/MS analysis of bistratamide N (**2**) by Marfey´s method using hydrolysis and derivatization with L-FDAA……………………………..…….. S20

Figure S32-S33. NMR spectra data of bistratamide K (3) in CD_3CN after addition of a $ZnCl_2$ solution: 0, 1, 2, 3, and 4 eq…………………………S21
Figure S34. (+)-ESI-TOFMS of bistratamide K (**3**) after addition of 4 equiv. of a $ZnCl_2$ solution……………….………………………………………S22

Table 1. NMR data of **1** and **2** in $CDCl_3$ and **3** in CD_3CN (500 MHz for ^1H and 125 MHz for ^{13}C)

	Bistratamide M (**1**)		Bistratamide N (**2**)		Bistratamide K (**3**)	
Position	δ_C, type	δ_H mult, (*J* in Hz)	δ_C type	δ_H mult, (*J* in Hz)	δ_C type	δ_H mult, (*J* in Hz)
1	159.7, C		159.0 , C		170.6 , C	
2	135.5 , C		135.6 , C		74.5 , CH	4.19, dd (7.2, 2.0)
3	141.9 , CH	8.27, s	141.5 , CH	8.23, s	81.6 , CH	4.83, dq (7.2, 6.7)
4	164.3 , C		164.6 , C		24.3 , CH_3	1.53, d (6.7)
5	44.2 , CH	5.38, m	44.1 , CH	5.37, m	168.5 , C	
6	19.9 , CH_3	1.72, d (7.1)	20.8 , CH_3	1.72, d (6.8)	52.3 , CH	4.62, ddd (9.3 3.0, 2.0)
7	159.4 , C		159.5 , C		32.1 , CH	2.05, m
8	149.2 , C		149.1 , C		15.8 , CH_3	0.40, d (6.9)
9	123.0 , CH	8.12, s	123.3 , CH	8.12, s	19.0 , CH_3	0.69, d (6.9)
10	167.1 , C		167.9 , C		170.7 , C	
11	55.3 , CH	5.44, m	54.9 , CH	5.54, m	78.6 , CH	5.01, dd (9.7, 8.1)
12	40.1 , CH	2.18, m	41.5 , CH	2.09 m	37.8 , CH2	3.63, m; 3.71, m
13	26.3 , CH_2	1.63, m; 1.24, m	25.6 , CH_2	1.67, m; 1.32, m	174.6 , C	
14	11.5 , CH3	1.01, t (7.4)	11.6 , CH_3	1.02, t (7.4)	53.7 , CH	5.25, m
15	14.5 , CH_3	0.87, d (6.8)	15.1 , CH_3	0.97, d (6.8)	40.4 , CH_2	3.12, m ; 3.31, m
16	159.8 , C		159.8 , C		136.8 , C	
17	148.2 , C		148.6 , C		130.8 , CH	7.18, m
18	125.0 , CH	8.22, s	124.3 , CH	8.17, s	129.1 , CH	7.25, m
19	171.6 , C		171.0 , C		127.9 , CH	7.24, m
20	48.2 , CH	5.40, m	47.7 , CH	5.46, m	129.1 , CH	7.25, m
21	23.9 , CH_3	1.74, d (6.9)	24.8 , CH_3	1.75, d (6.7)	130.8 , CH	7.18, m
22					160.0 , C	
23					149.8 , C	
24					125.0 , CH	8.06, s
25					172.6 , C	
26					48.0 , CH	5.25, m
27					24.3 , CH_3	1.52, d (6.7)
NH-1		8.69, d (5.7)		8.71, d (6.5)		7.86, d (7.5)
NH-2		8.64, d (7.2)		8.65, d (7.3)		7.19, d (9.6)
NH-3		8.42, d (8.0)		8.46, d (9.0)		8.12, d (8.1)

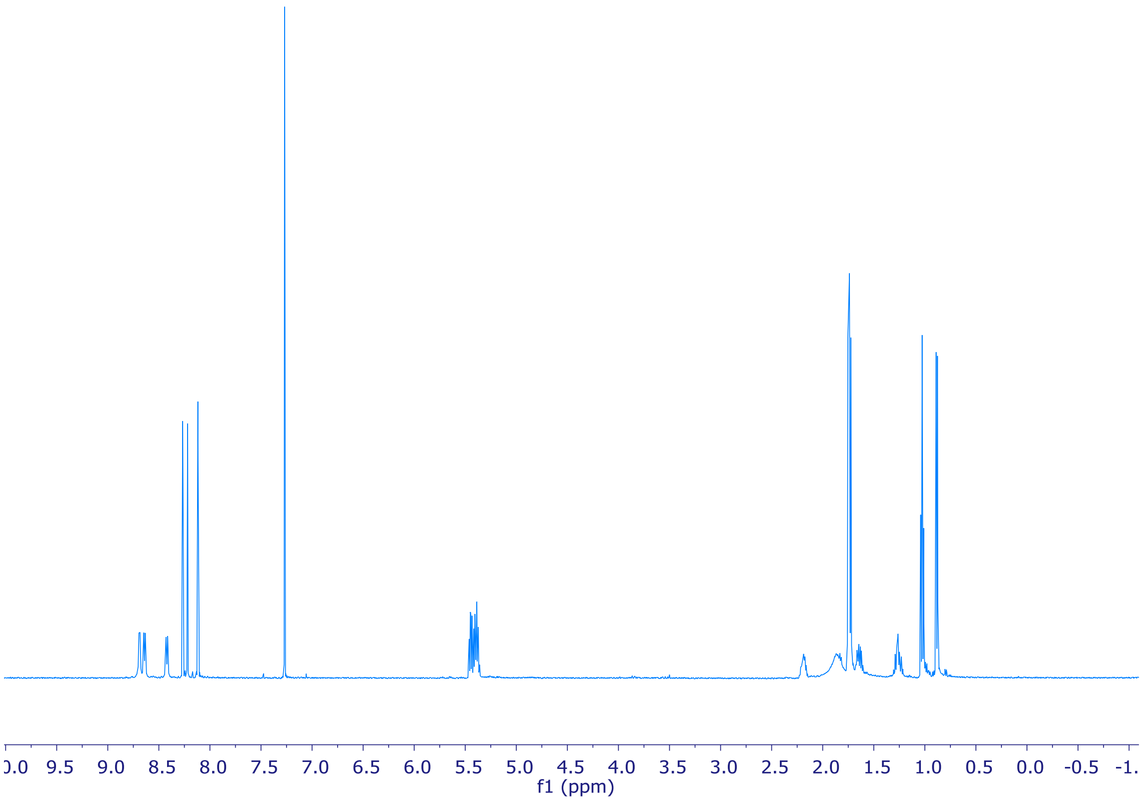

Figure S1. ^{1}H NMR spectrum of bistratamide M (**1**) (500 MHz, $CDCl_3$).

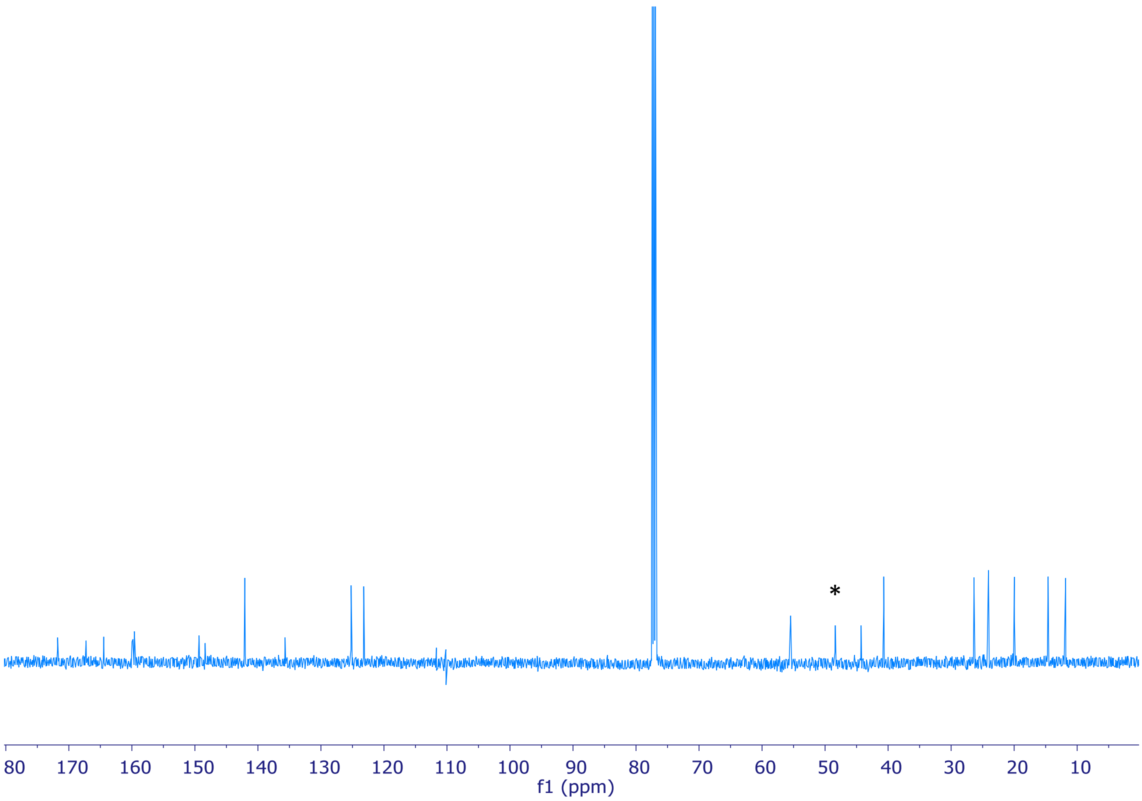

Figure S2. ^{13}C NMR spectrum of bistratamide M (**1**) (125 MHz, $CDCl_3$). * MeOH traces.

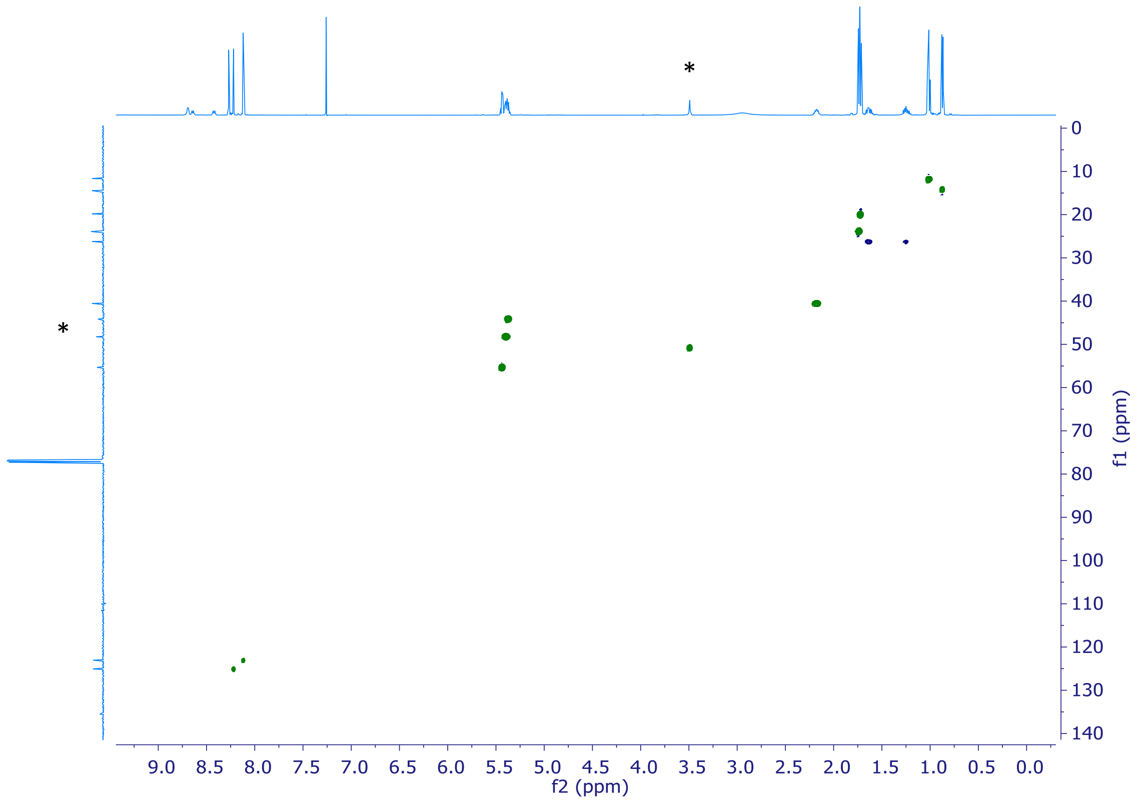

Figure S3. *g*-HSQC spectrum of bistratamide M (**1**). * MeOH traces

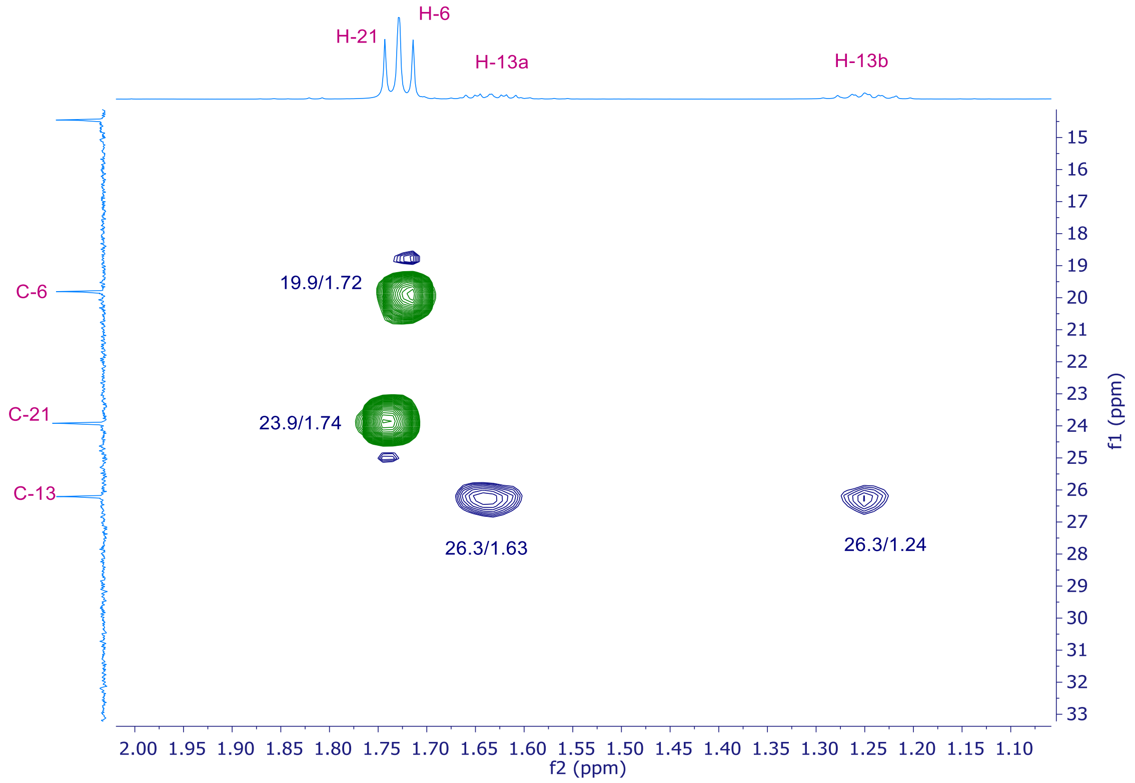

Figure S4. Expanding g-HSQC spectrum of bistratamide M (**1**).

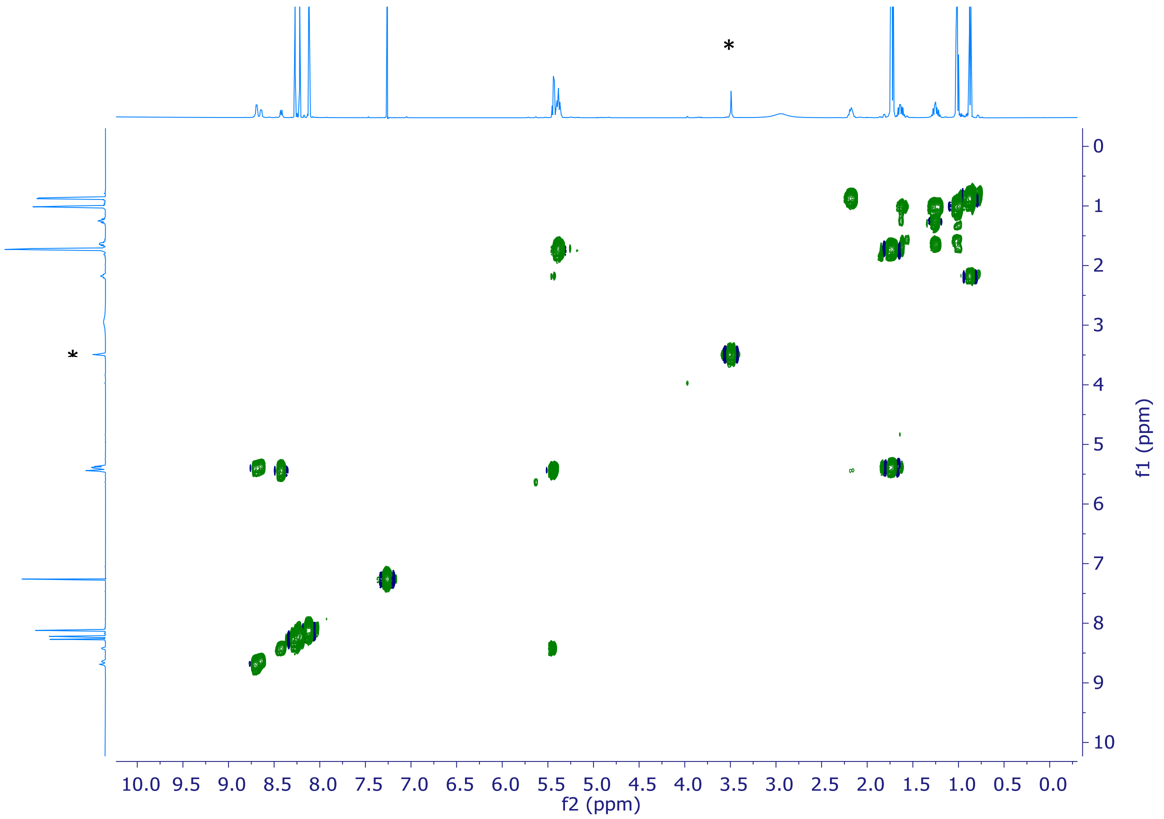

Figure S5. *g*-COSY spectrum of bistratamide M (**1**). * MeOH traces

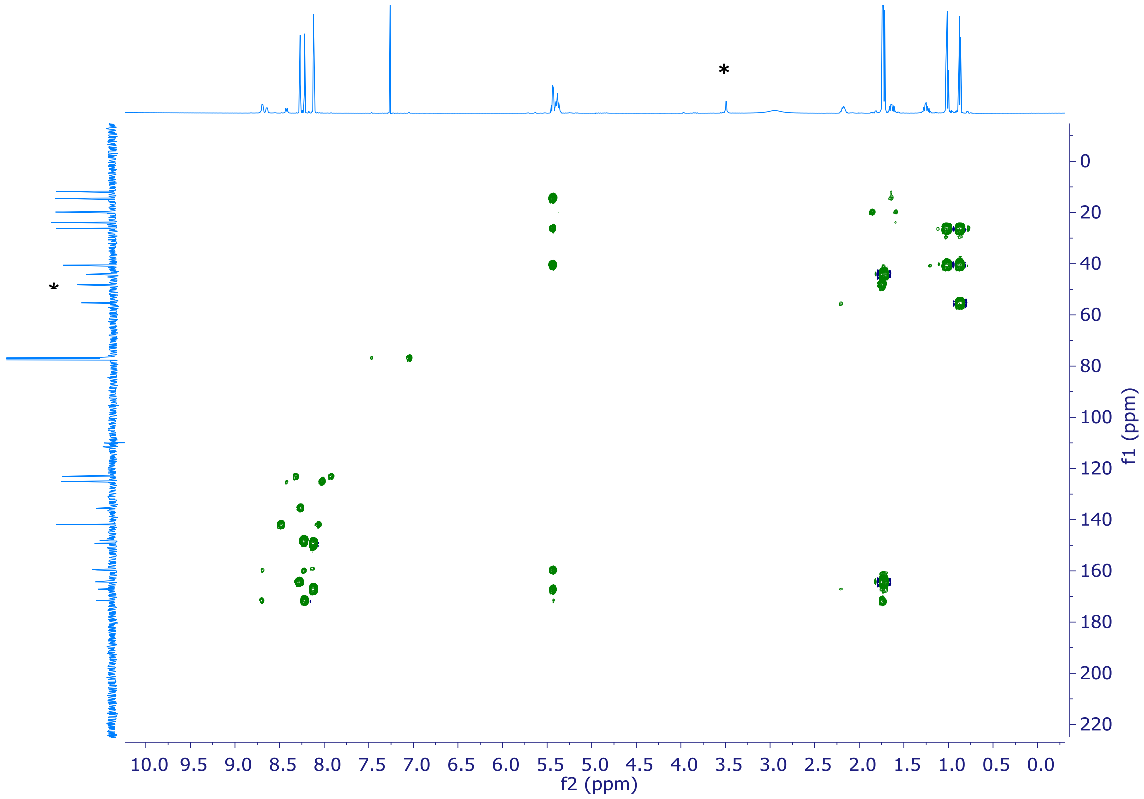

Figure S6. *g*-HMBC spectrum of bistratamide M (**1**). * MeOH traces

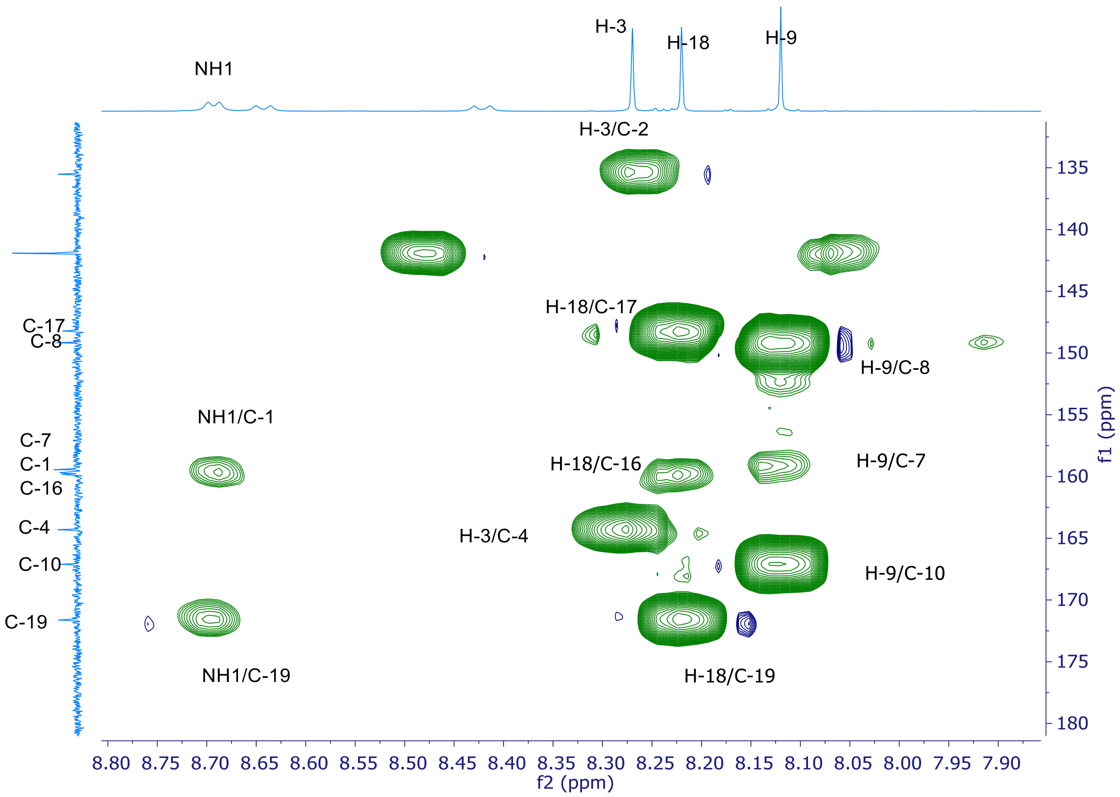

Figure S7. Expanding g-HMBC spectrum of bistratamide M (**1**).

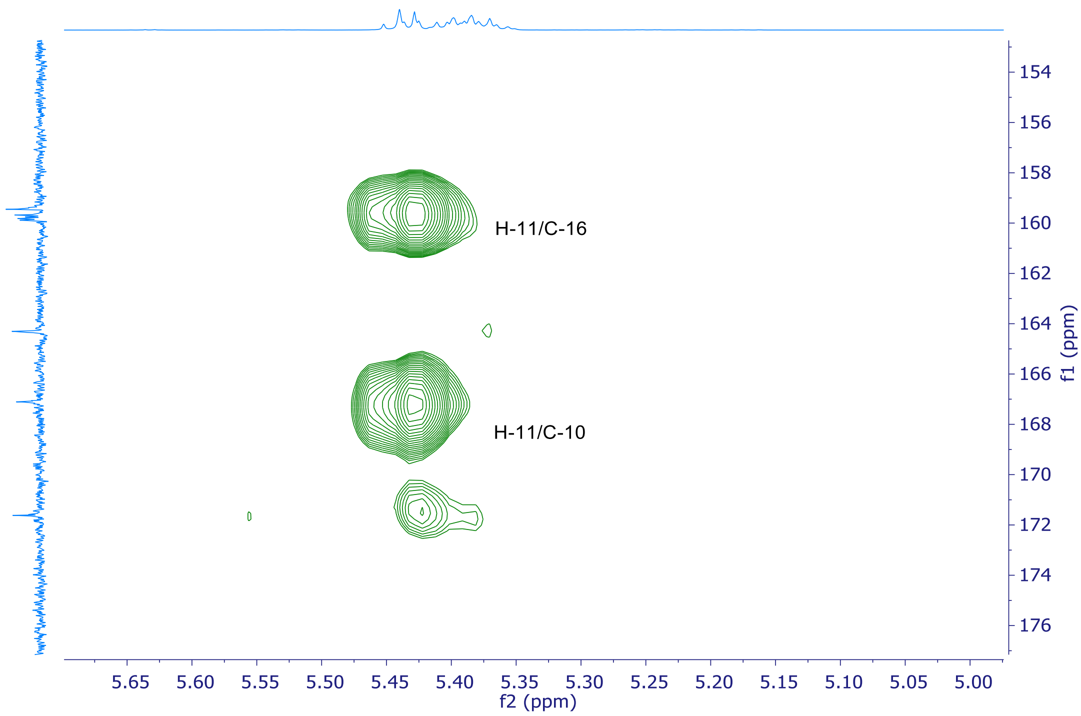

Figure S8. Expanding g-HMBC spectrum of bistratamide M (**1**).

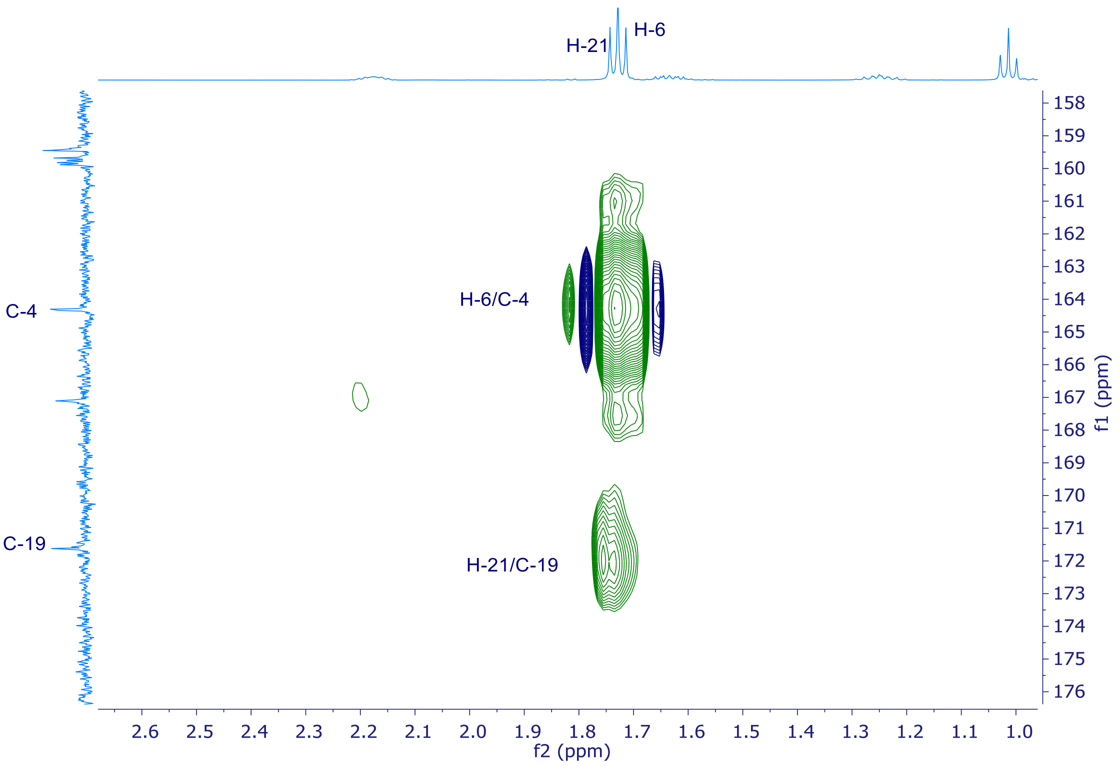

Figure S9. Expanding g-HMBC spectrum of bistratamide M (**1**).

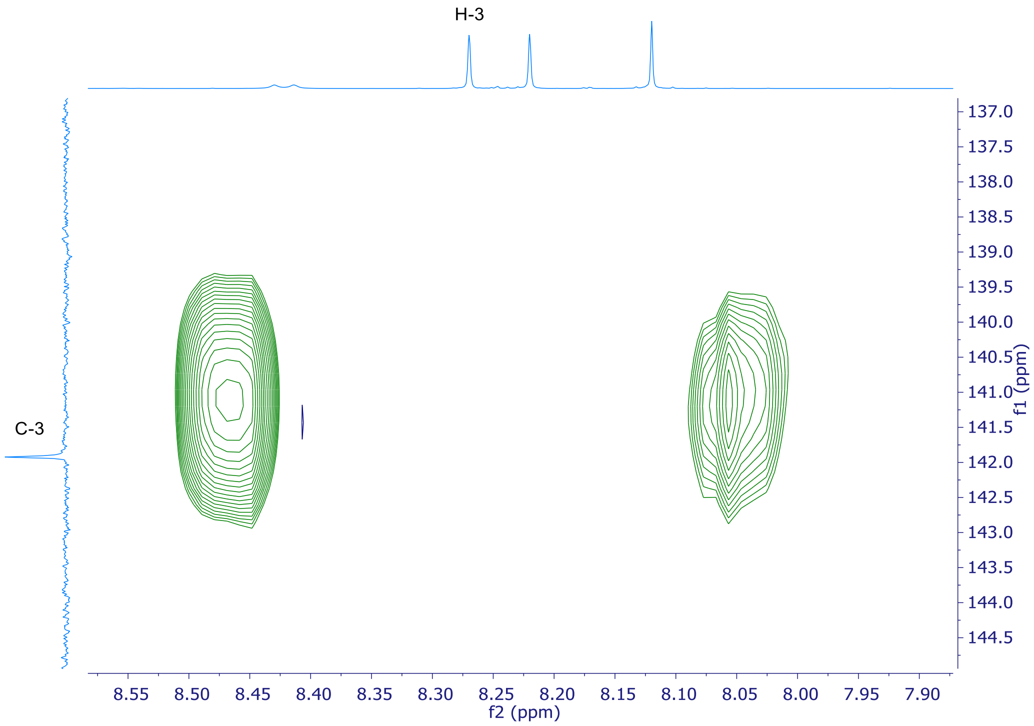

Figure S10. Expanding g-HMBC spectrum of bistratamide M (**1**).

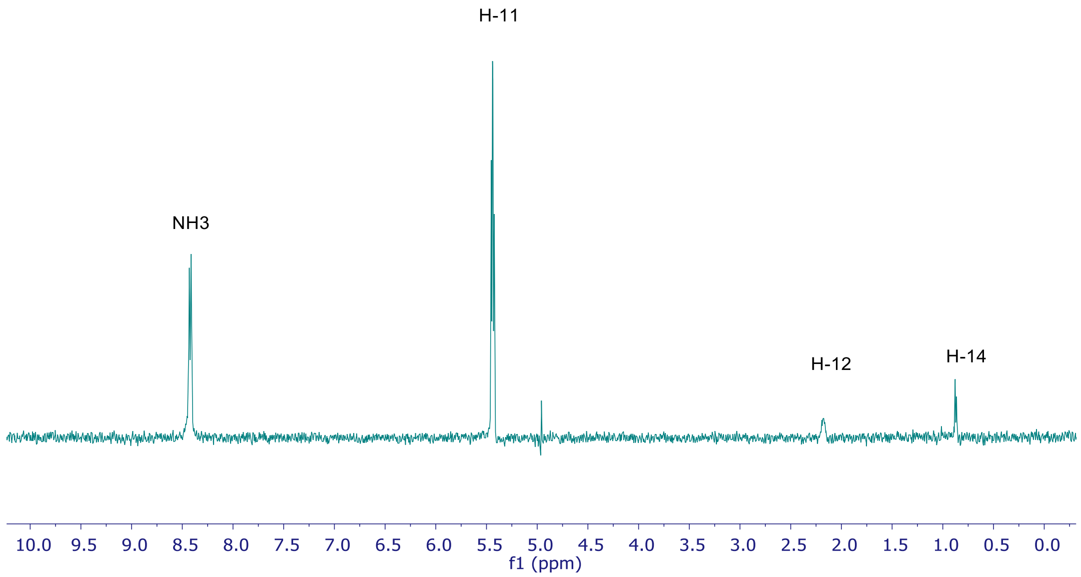

Figure S11. Selective TOCY spectrum at 8.42 ppm (NH-3) of bistratamide M (**1**).

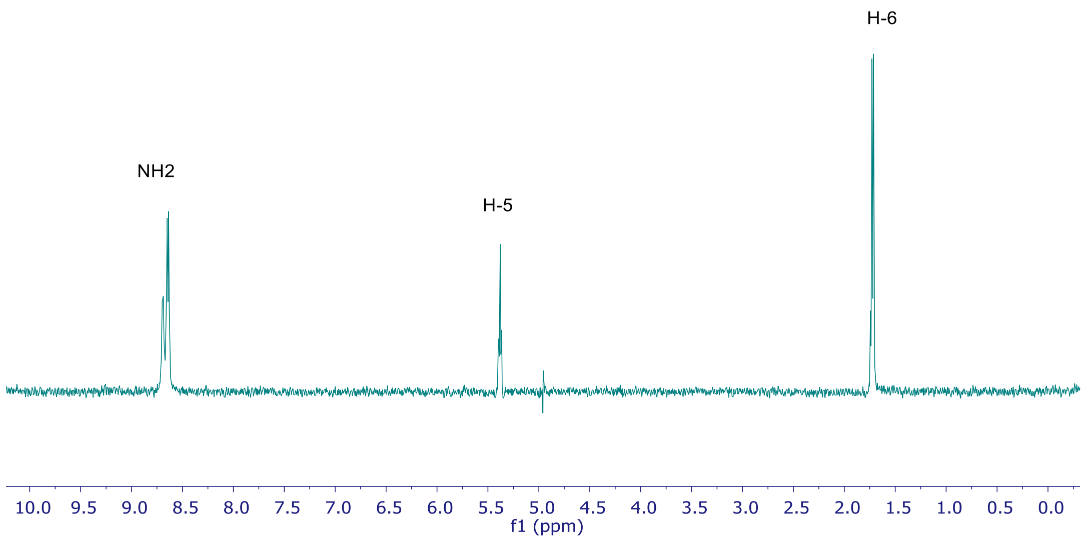

Figure S12. Selective TOCY spectrum at 8.64 ppm (NH-2) of bistratamide M (**1**).

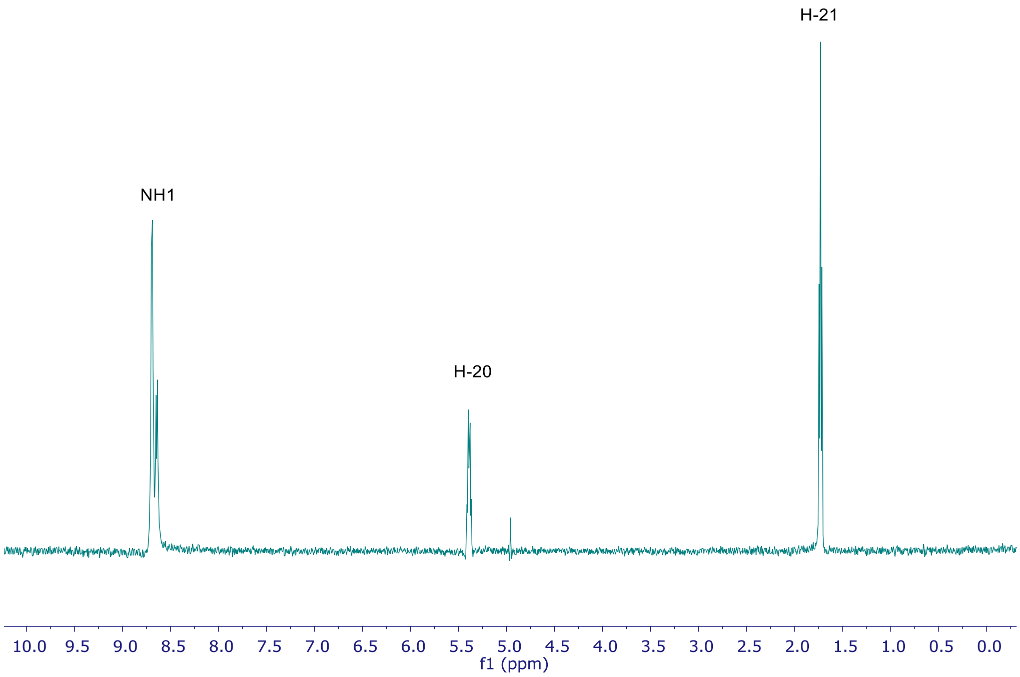

Figure S13. Selective TOCY spectrum at 8.69 ppm (NH-1) of bistratamide M (**1**)

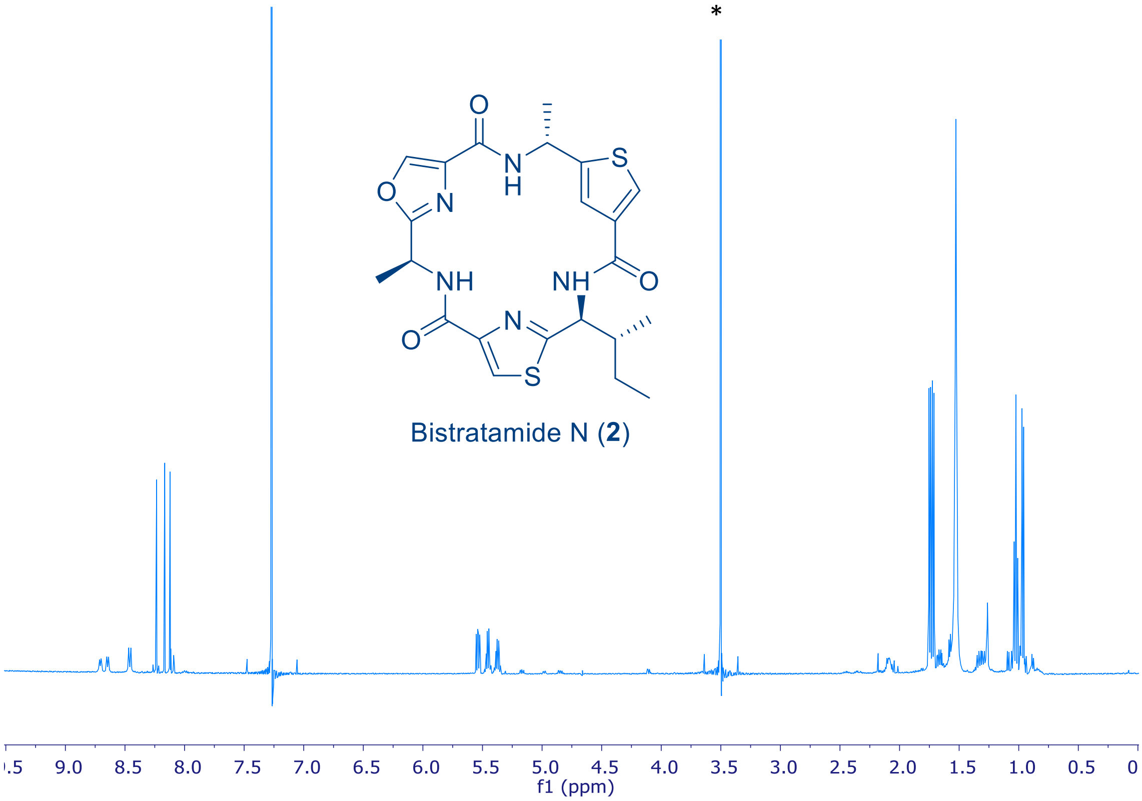

Figure S14 ^{1}H NMR spectrum of bistratamide N (**2**) (500 MHz, $CDCl_3$). *MeOH traces.

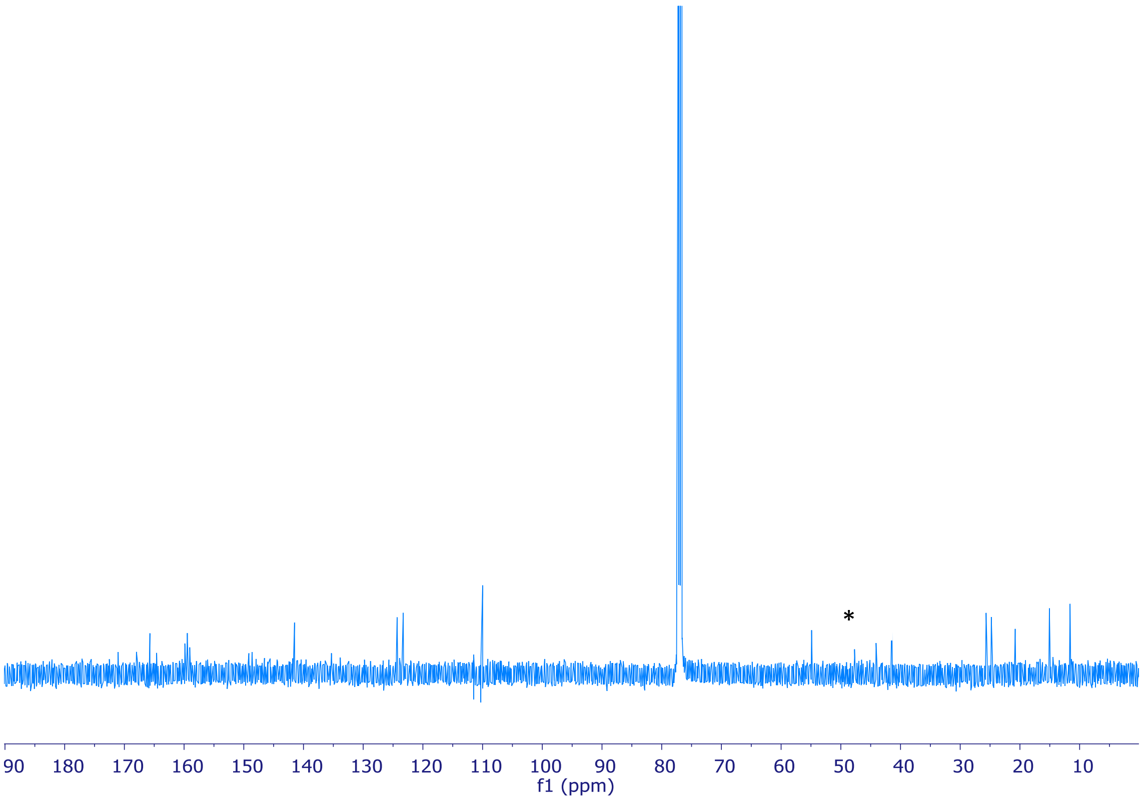

Figure S15. ^{13}C NMR spectrum of bistratamide N (**2**) (125 MHz, $CDCl_3$). *MeOH traces

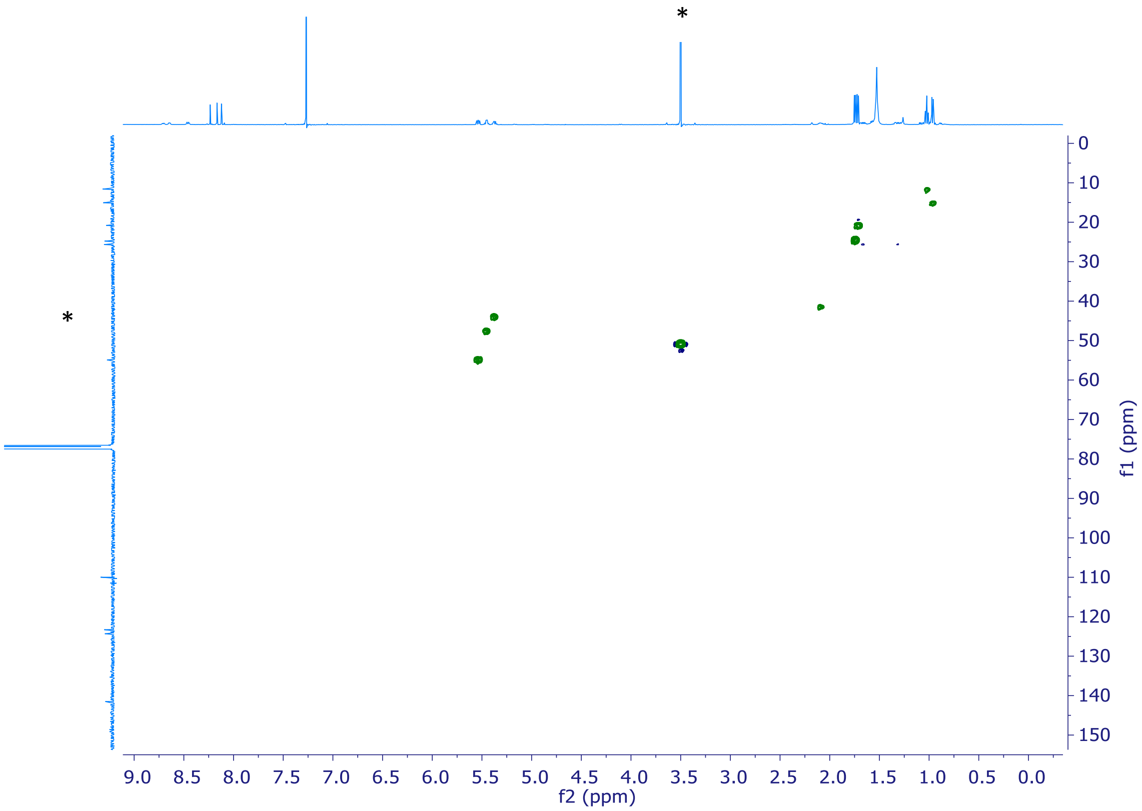

Figure S16. *g*-HSQC spectrum of bistratamide N (**2**). *MeOH traces

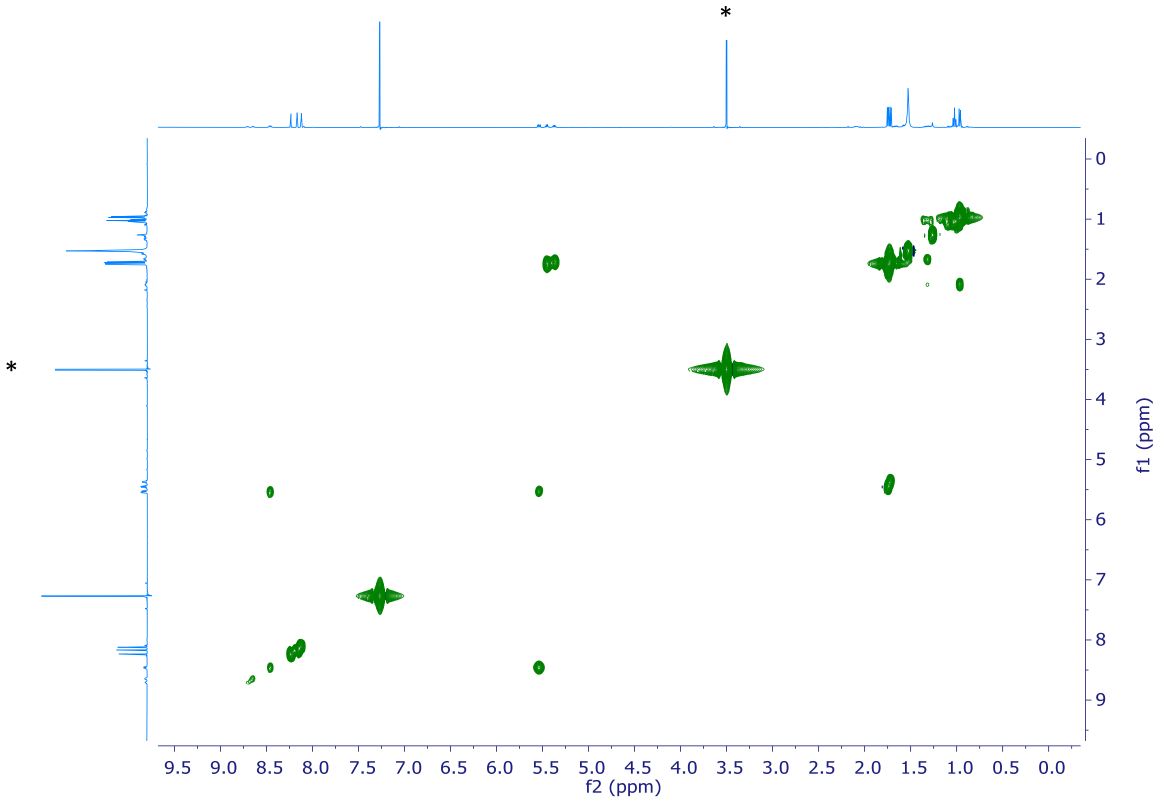

Figure S17. *g*-COSY spectrum of bistratamide N (**2**). *MeOH traces

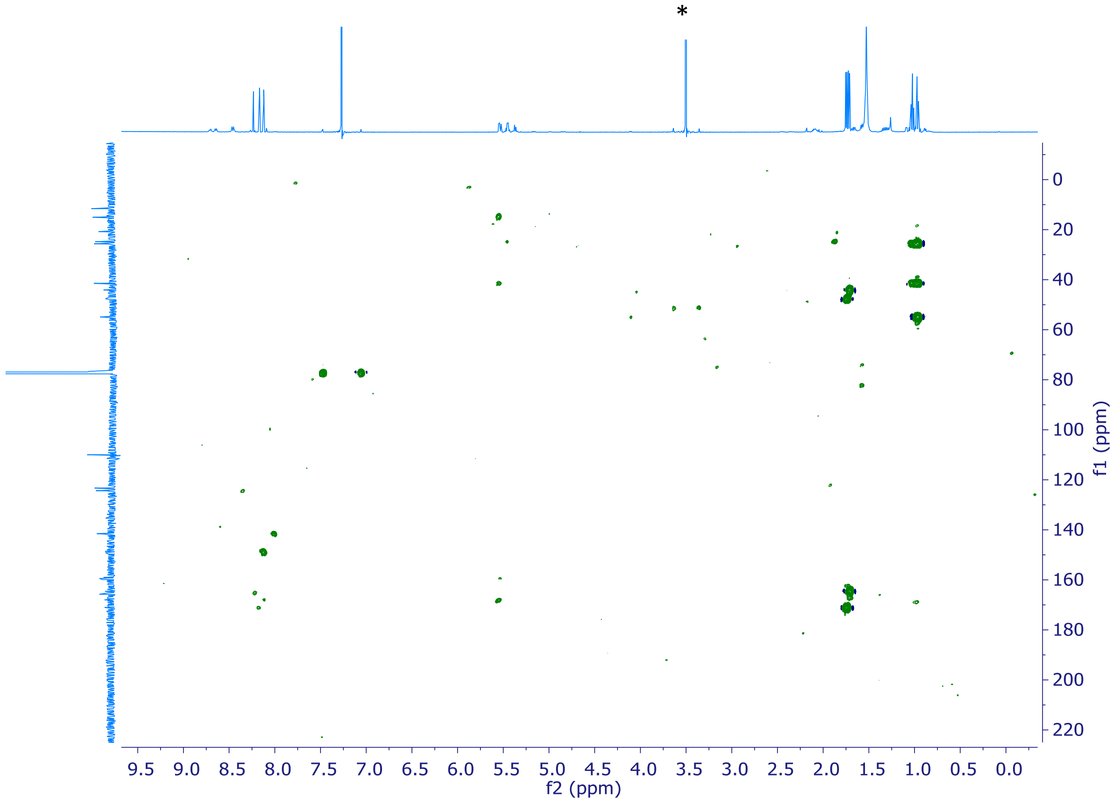

Figure S18. *g*-HMBC spectrum of bistratamide N (**2**). *MeOH traces

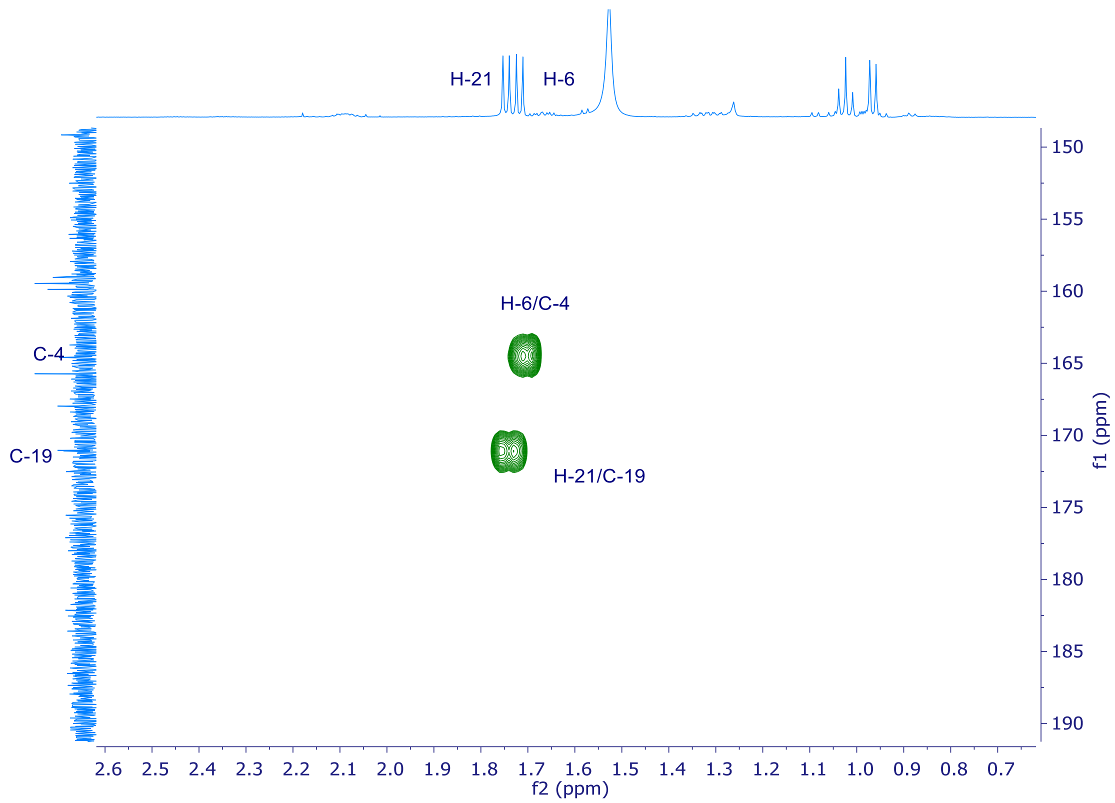

Figure S19. Expanding *g*-HMBC spectrum of bistratamide N (**2**).

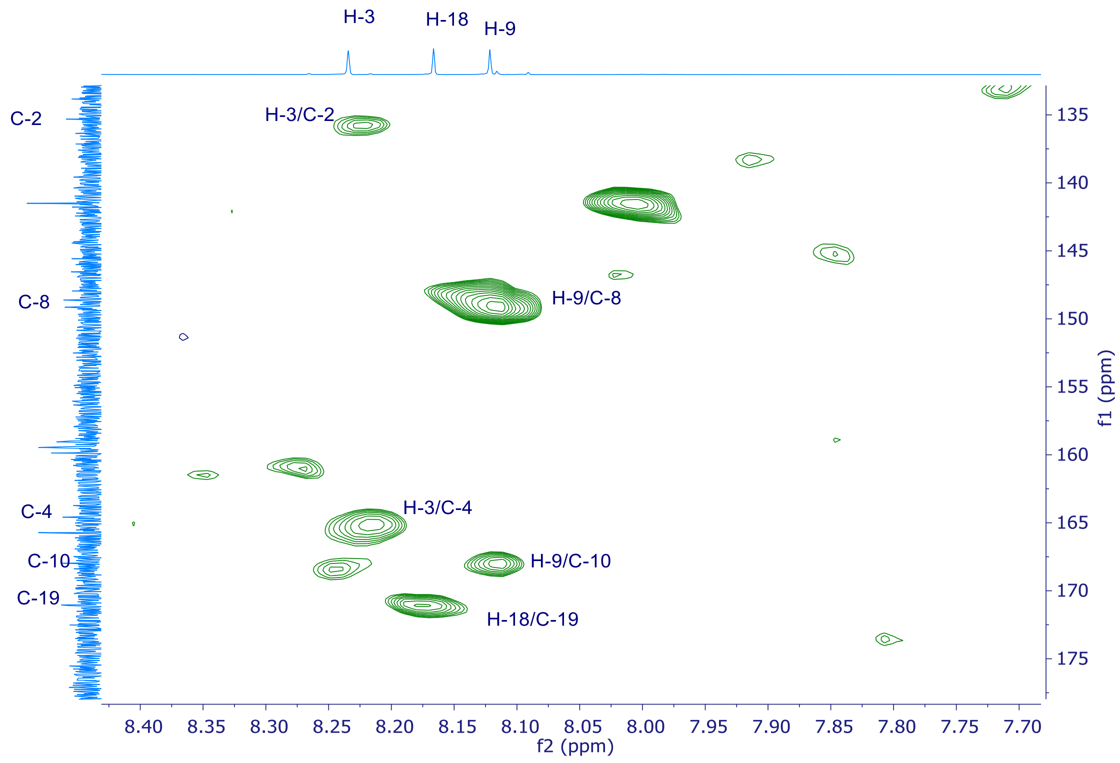

Figure S20. Expanding *g*-HMBC spectrum of bistratamide N (**2**).

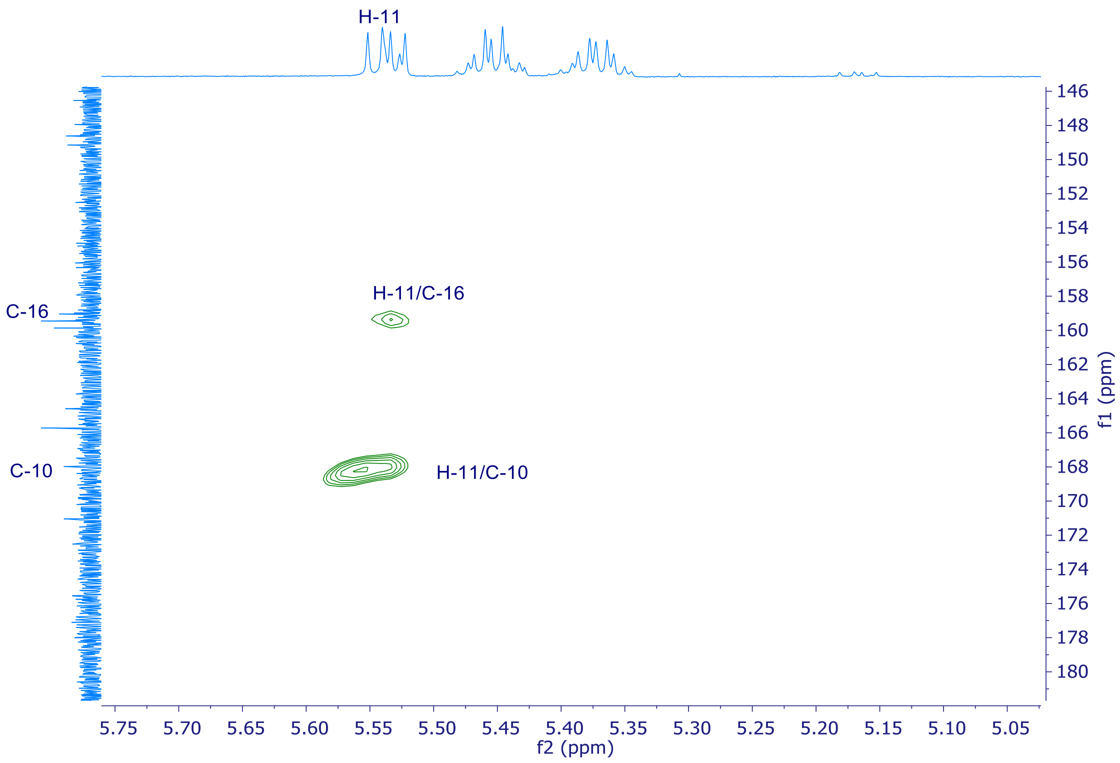

Figure S21. Expanding *g*-HMBC spectrum of bistratamide N (**2**).

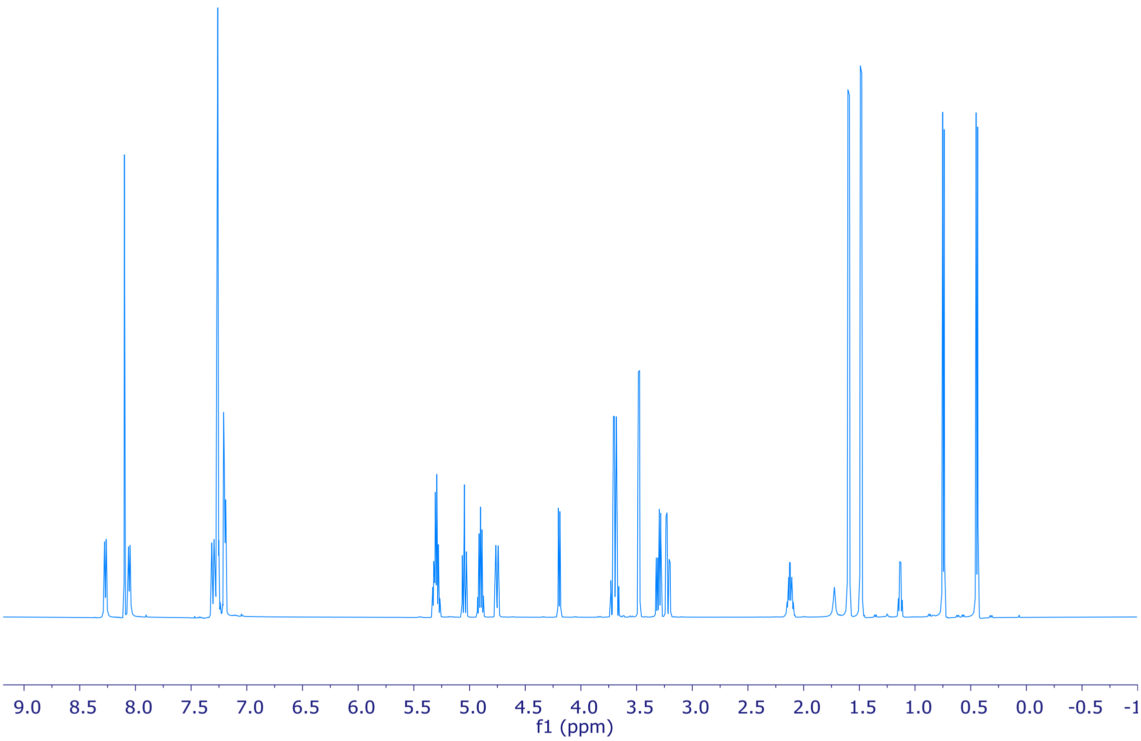

Figure S22. ^{1}H NMR spectrum of bistratamide K (**3**) (500 MHz, $CDCl_3$).

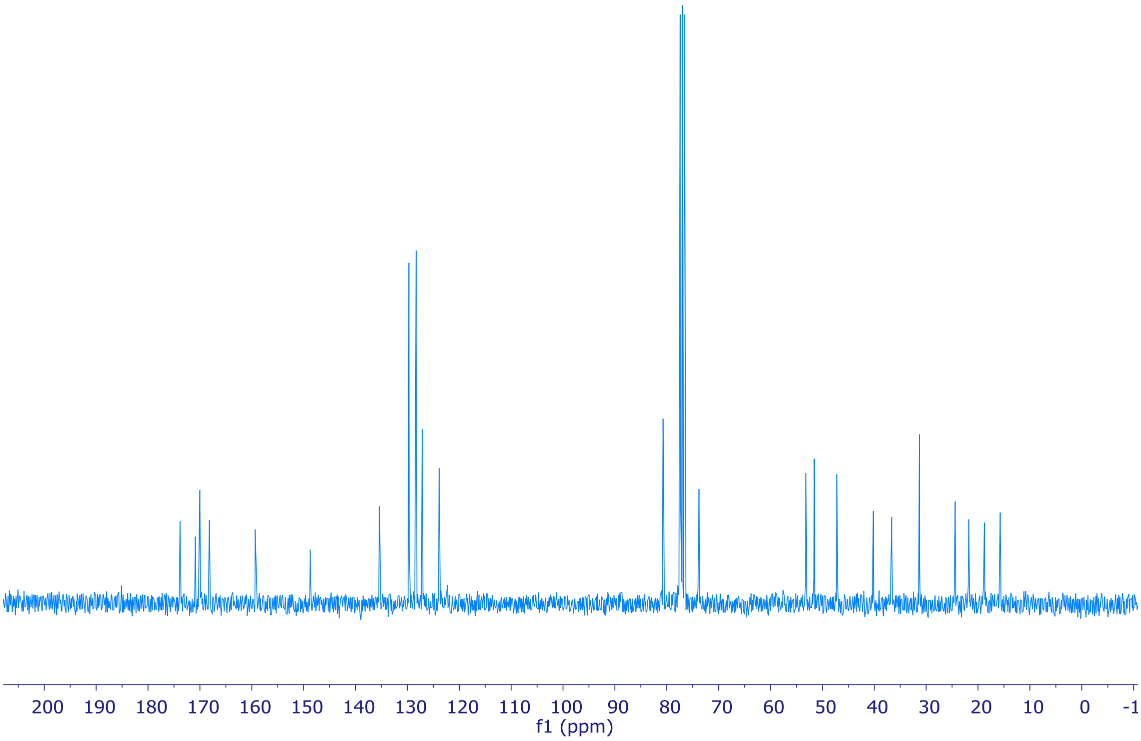

Figure S23. ^{13}C NMR spectrum of bistratamide K (**3**) (500 MHz, $CDCl_3$).

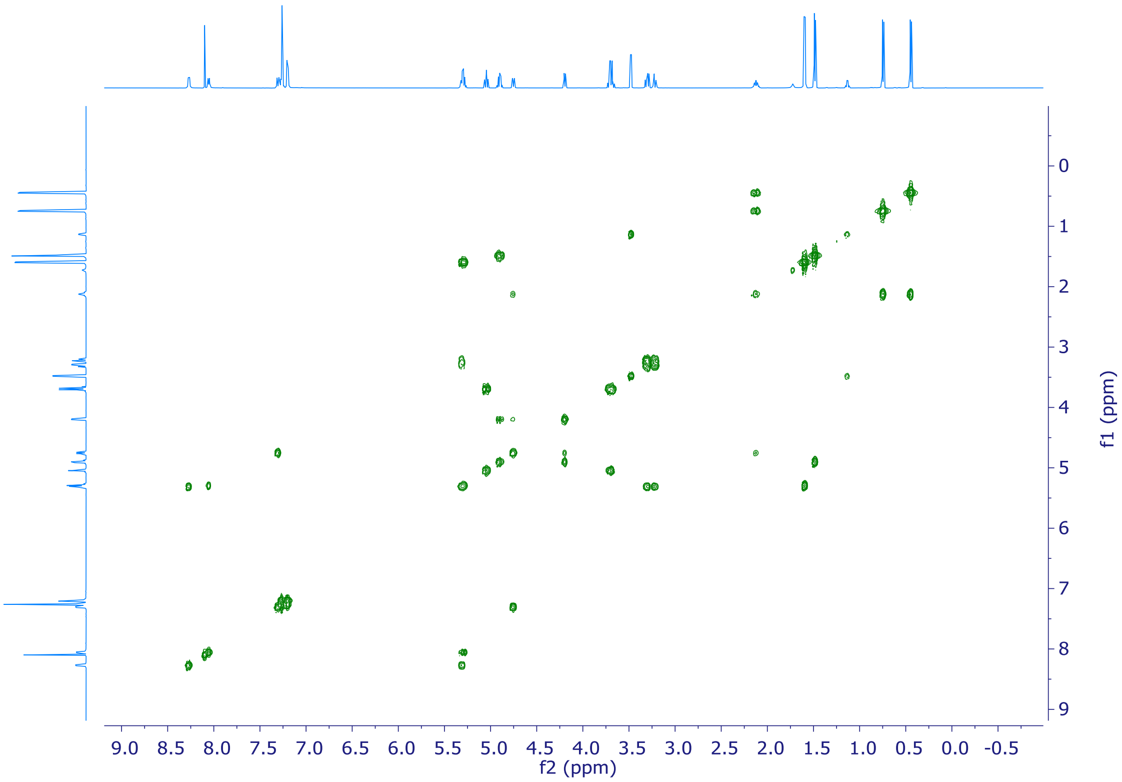

Figure S24. *g*-COSY spectrum of bistratamide K (**3**).

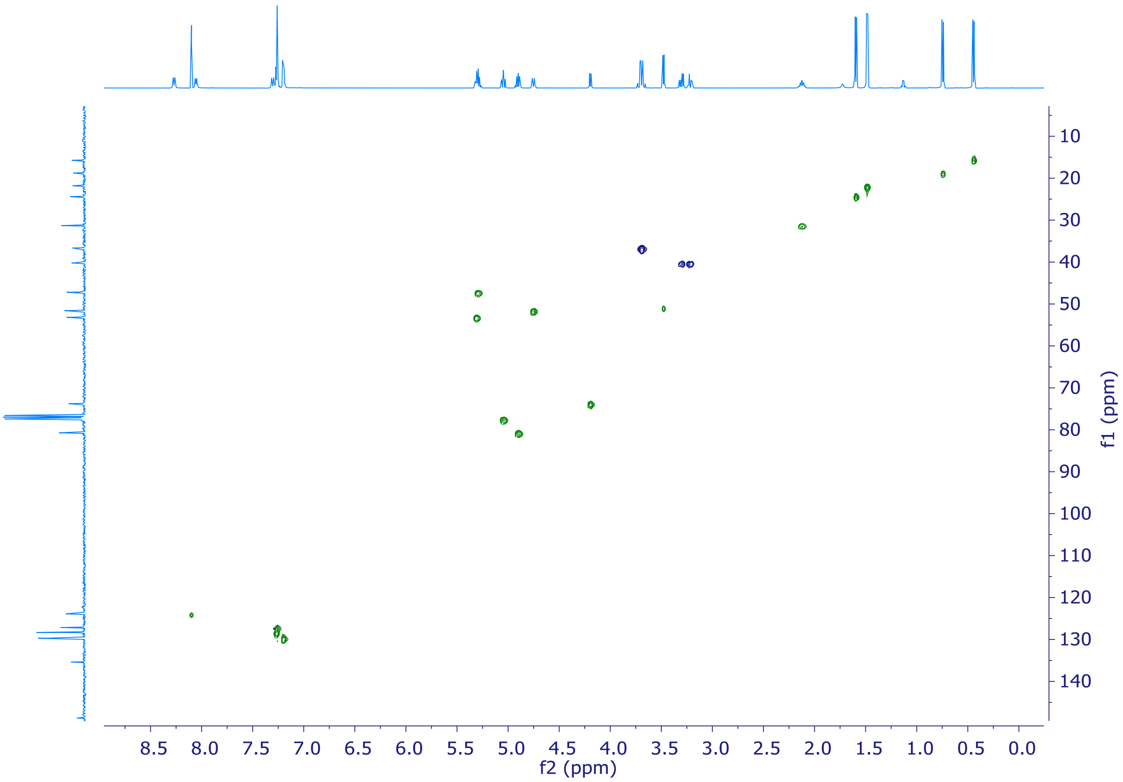

Figure S25. *g*-HSQC spectrum of bistratamide K (**3**).

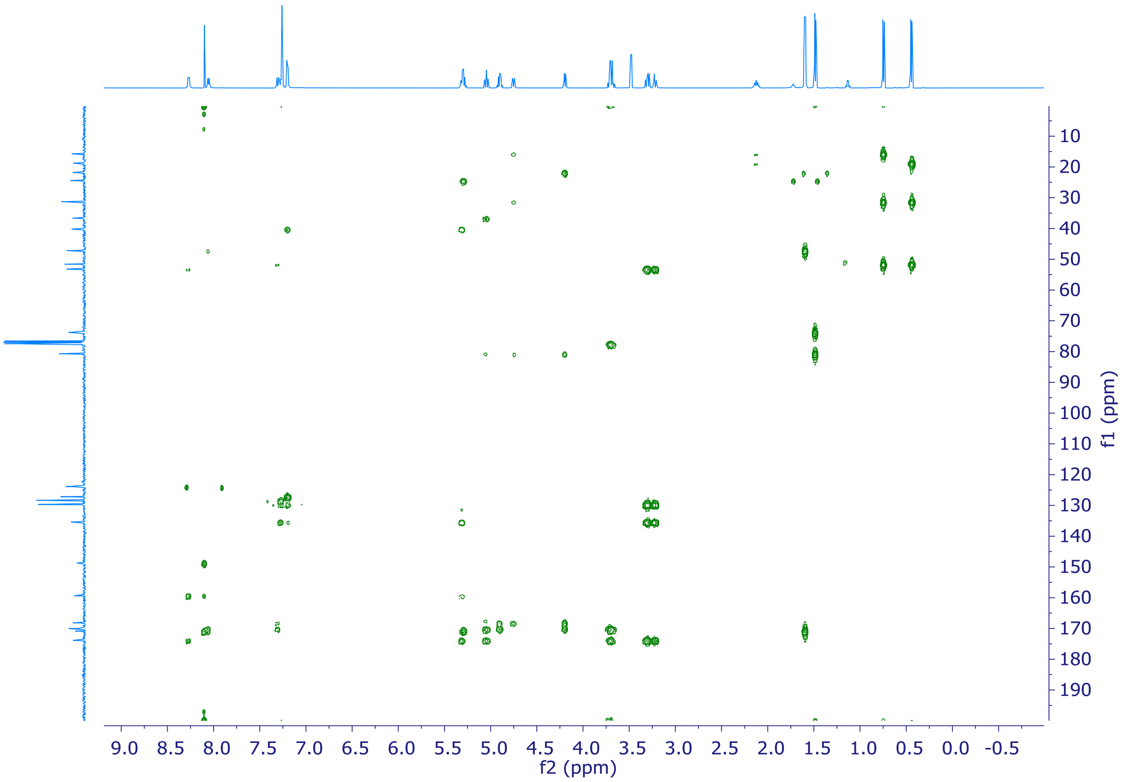

Figure S26. *g*-HMBC spectrum of bistratamide K (**3**).

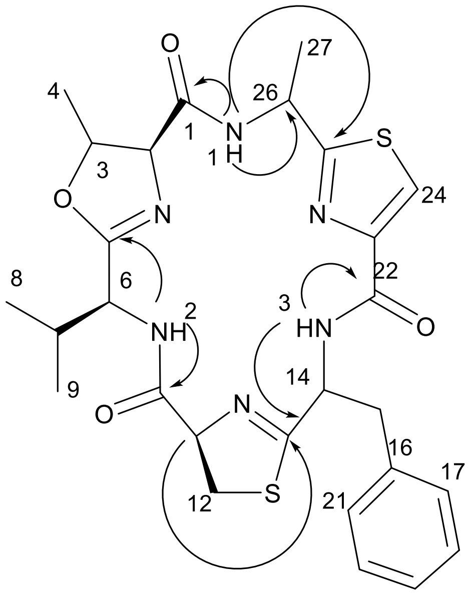

Figure S27. Structure and key HMBC of bistratamide K (**3**).

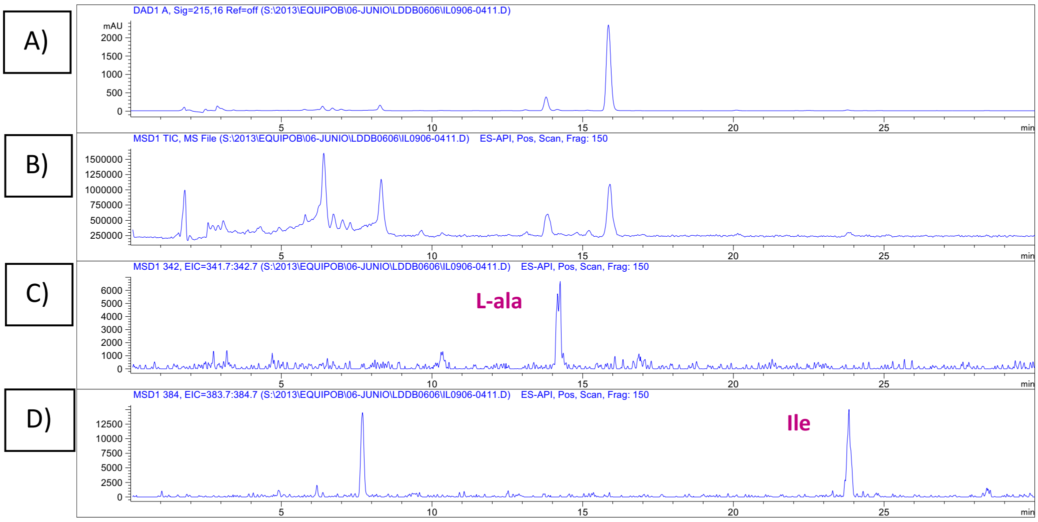

Figure S28. LC/MS analysis of bistratamide M (**1**) by Marfey´s method using ozonolysis, hydrolysis and derivatization with L-FDAA: A) Total ion current (TIC) chromatogram. B) MS chromatogram. C) Extracted mass chromatogram from ion extraction at *m/z* 342. D) Extracted mass chromatogram from ion extraction at *m/z* 384.

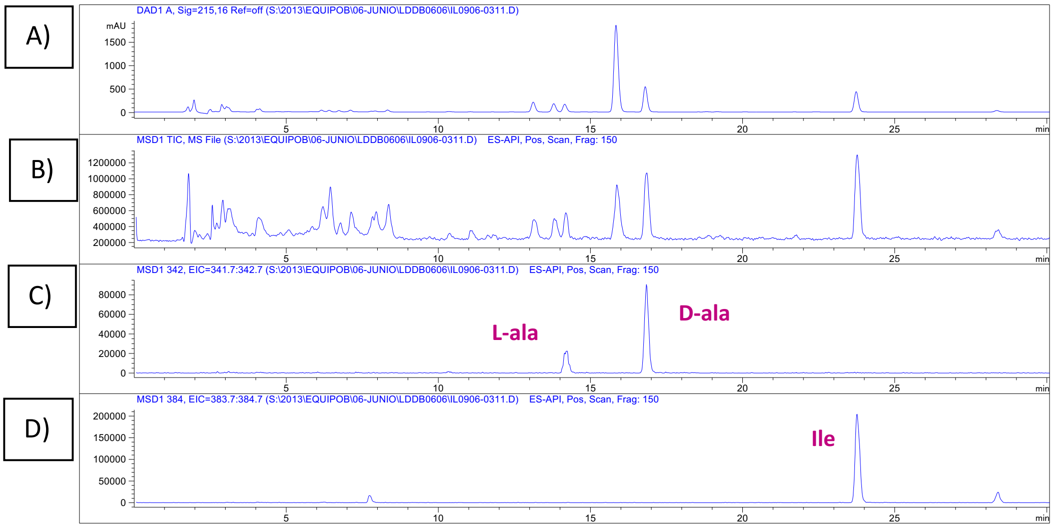

Figure S29. LC/MS analysis of bistratamide N (**2**) by Marfey´s method using ozonolysis, hydrolysis and derivatization with L-FDAA: A) Total ion current (TIC) chromatogram. B) MS chromatogram. C) Extracted mass chromatogram from ion extraction at *m/z* 342. D) Extracted mass chromatogram from ion extraction at *m/z* 384.

.

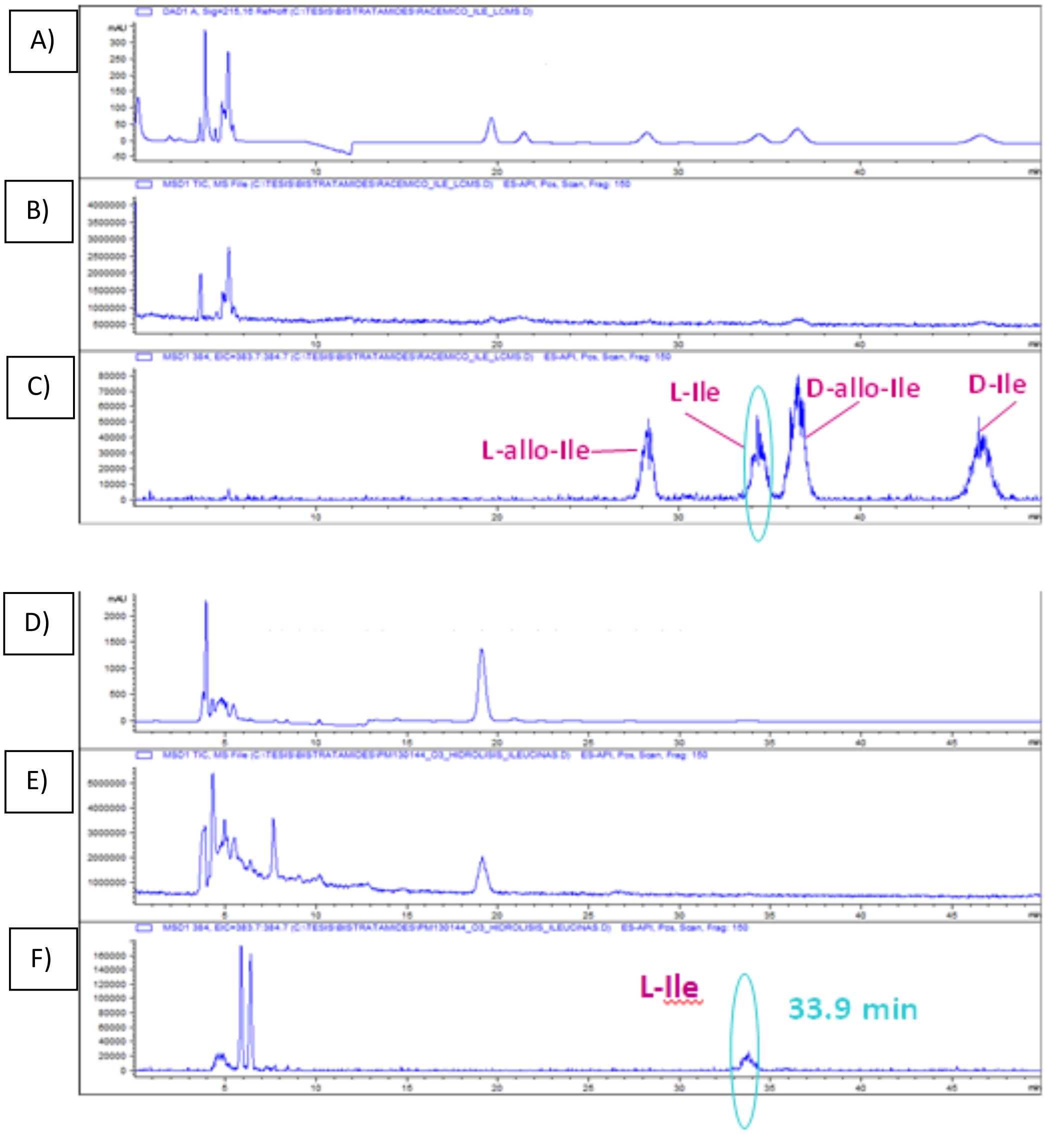

Figure S30. Analysis of bistratamide M (**1**) by Advance Marfey´s method:

1.- LC/MS analysis of racemic isoleucine with L+D-FDAA: A) Total ion current (TIC) chromatogram. B) MS chromatogram. C) Extracted mass chromatogram from ion extraction at *m/z* 384.

2.- LC/MS analysis of bistratamide M (**1**) with L-FDAA: D) Total ion current (TIC) chromatogram. E) MS chromatogram. F) Extracted mass chromatogram from ion extraction at *m/z* 384.

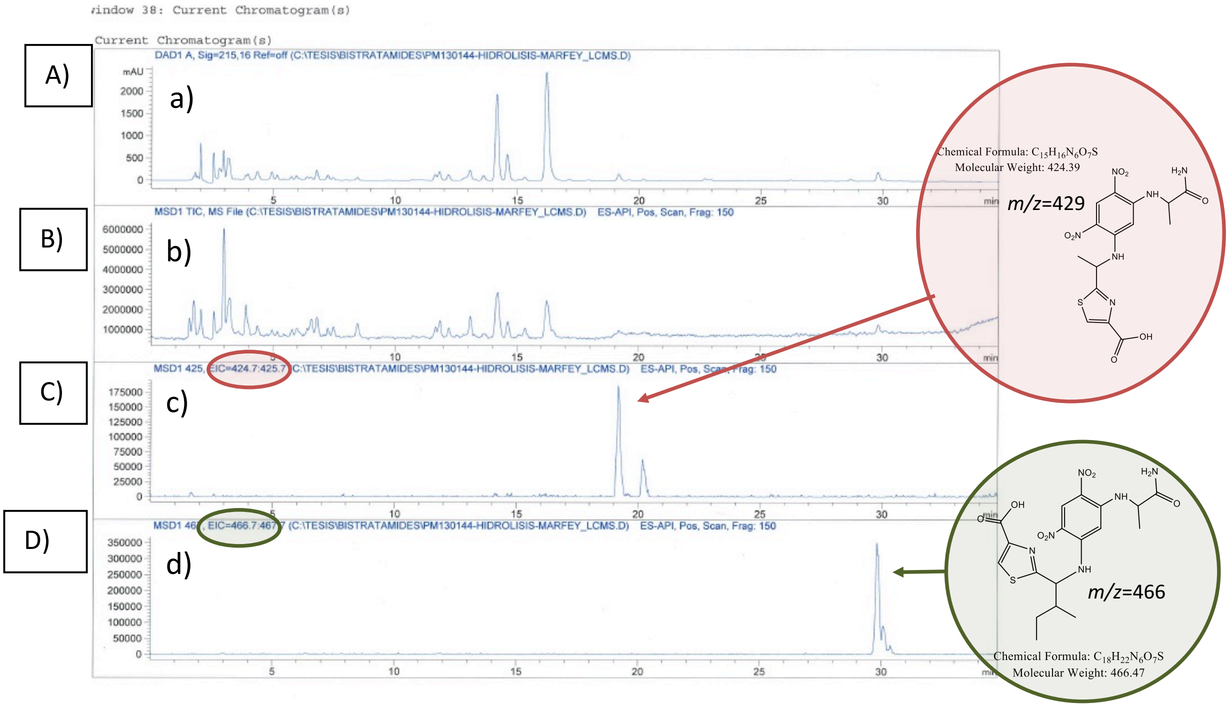

Figure S31. LC/MS analysis of bistratamide N (**2**) by Marfey´s method using hydrolysis and derivatization with L-FDAA: A) Total ion current (TIC) chromatogram. B) MS chromatogram. C) Extracted mass chromatogram from ion extraction at *m/z* 429. D) Extracted mass chromatogram from ion extraction at *m/z* 466.

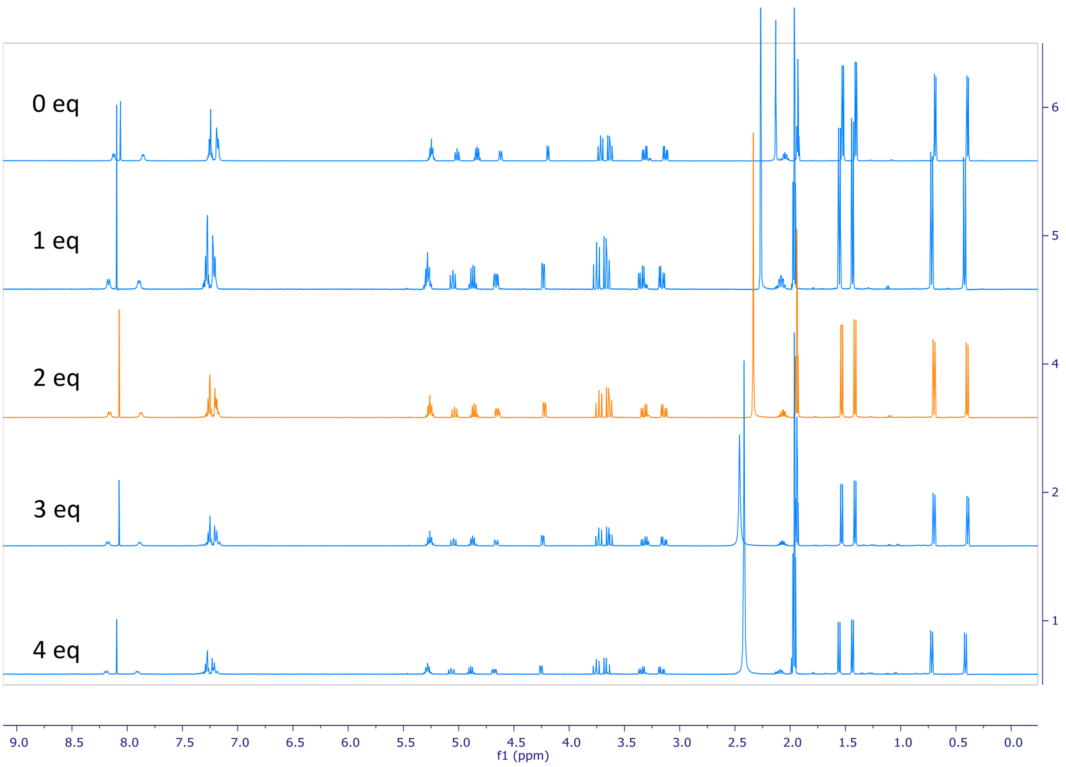

Figure S32. ^{1}H NMR spectra in CD_3CN of compound **3** after addition of a $ZnCl_2$ solution: 0, 1, 2, 3, and 4 eq.

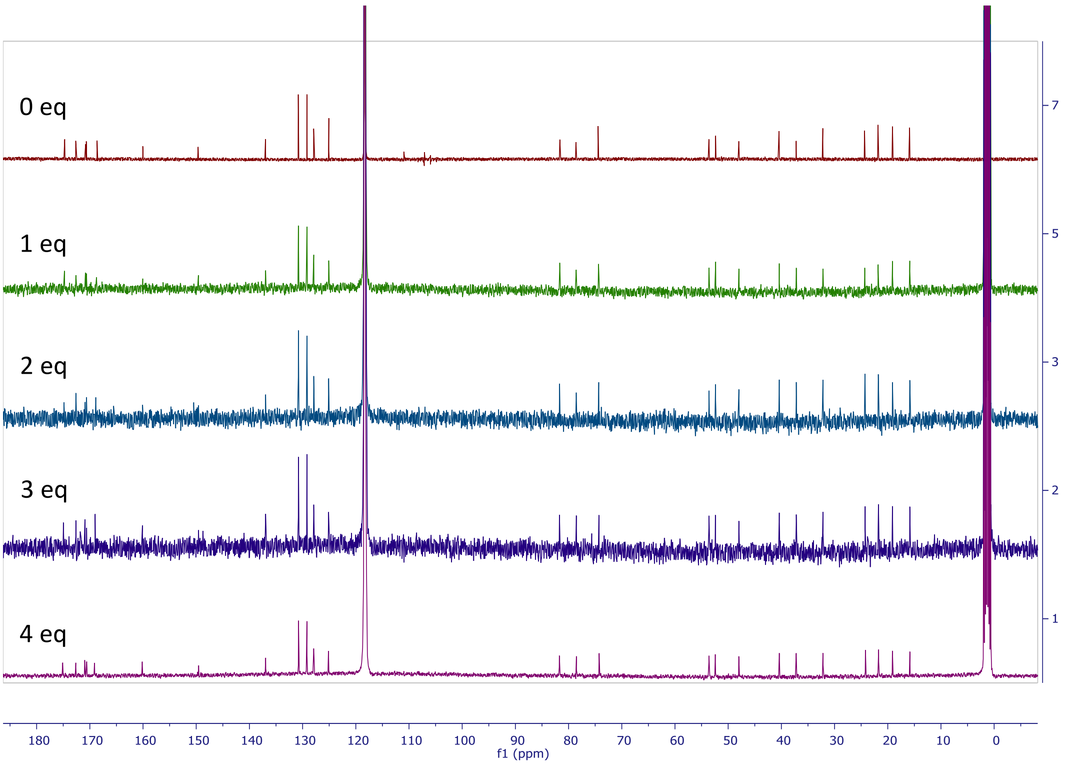

Figure S33. ^{13}C NMR spectra in CD_3CN of compound **3** after addition of a $ZnCl_2$ solution: 0, 1, 2, 3, and 4 eq.

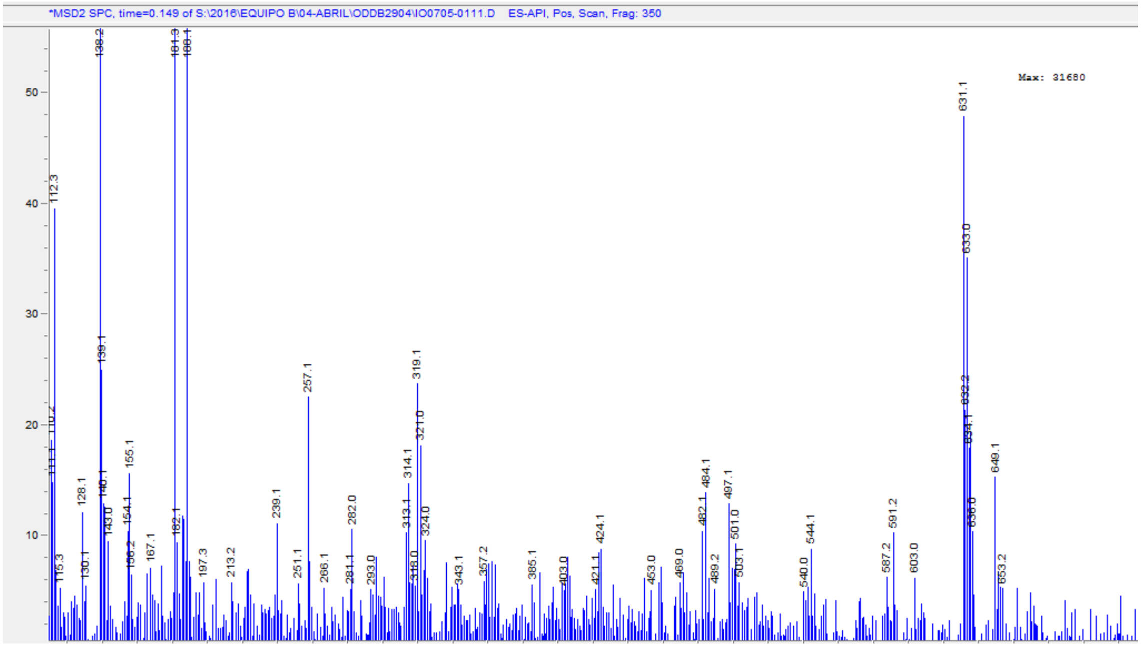

Figure S34. (+)-LRESI-TOF mass spectrum of **3** after addition of 4 equiv. of a $ZnCl_2$ solution.
